# Unsupervised Learning in an Ensemble of Spiking Neural Networks Mediated by ITDP

**DOI:** 10.1371/journal.pcbi.1005137

**Published:** 2016-10-19

**Authors:** Yoonsik Shim, Andrew Philippides, Kevin Staras, Phil Husbands

**Affiliations:** 1 Centre for Computational Neuroscience and Robotics, University of Sussex, Falmer, Brighton, United Kingdom; 2 Neuroscience, School of Life Sciences, University of Sussex, Falmer, Brighton, United Kingdom; University College London, UNITED KINGDOM

## Abstract

We propose a biologically plausible architecture for unsupervised ensemble learning in a population of spiking neural network classifiers. A mixture of experts type organisation is shown to be effective, with the individual classifier outputs combined via a gating network whose operation is driven by input timing dependent plasticity (ITDP). The ITDP gating mechanism is based on recent experimental findings. An abstract, analytically tractable model of the ITDP driven ensemble architecture is derived from a logical model based on the probabilities of neural firing events. A detailed analysis of this model provides insights that allow it to be extended into a full, biologically plausible, computational implementation of the architecture which is demonstrated on a visual classification task. The extended model makes use of a style of spiking network, first introduced as a model of cortical microcircuits, that is capable of Bayesian inference, effectively performing expectation maximization. The unsupervised ensemble learning mechanism, based around such spiking expectation maximization (SEM) networks whose combined outputs are mediated by ITDP, is shown to perform the visual classification task well and to generalize to unseen data. The combined ensemble performance is significantly better than that of the individual classifiers, validating the ensemble architecture and learning mechanisms. The properties of the full model are analysed in the light of extensive experiments with the classification task, including an investigation into the influence of different input feature selection schemes and a comparison with a hierarchical STDP based ensemble architecture.

## Author Summary

Ensemble effects appear to be common in the nervous system. That is, there are many examples of where groups of neurons, or groups of neural circuits, act together to give better performance than is possible from a single neuron or single neural circuit. For instance, there is evidence that ensembles of spatially distinct neural circuits are involved in some classification tasks. Several authors have suggested that architectures for ensemble learning similar to those developed in machine learning and artificial intelligence might be active in the brain, coordinating the activity of populations of classifier circuits. However, to date it has not been clear what kinds of biologically plausible mechanism might underpin such a scheme. Our model shows how such an architecture can be successfully constructed though the use of the rather understudied mechanism of input timing dependent plasticity (ITDP) as a way of coordinating and guiding the activity of a population of model cortical microcircuits. The model is successfully demonstrated on a visual classification task (recognizing hand written integers).

## Introduction

There is growing evidence that many brain mechanisms involved in perception and learning make use of ensemble effects, whereby groups of neurons, or groups of neural circuits, act together to improve performance. At the lowest level of neuronal organisation it appears that the collective activity of groups of neurons is used to overcome the unreliable, stochastic nature of single neuron firing during the learning of motor skills [1, 2]. There are also many examples at higher levels of organisation. For instance Li et al. (2008) [3] used a combination of functional magnetic resonance imaging and olfactory psychophysics to show that initially indistinguishable odours become discriminable after aversive conditioning, and that during the learning process there were clear, spatially diverse ensemble activity patterns across the primary olfactory (piriform) cortex and in the orbitofrontal cortex. They hypothesized that in this case fear conditioning recruits functionally distinct networks from across the cortex which act in concert to maximize adaptive behaviour. Many others have suggested that the integration of information from multiple sensory modalities and different areas of the cortex, in complex recognition or other cognitive tasks, may involve ensemble learning mechanisms [4–9]. For instance, the influential ‘functional constancy’, or ‘metamodal’, theory of cortical operation [10, 11] suggests coordinated action of multiple areas during learning and cognitive processing [6]. The hypothesis is that different cortical areas have a core functional, or information processing, specialization, and this is maintained following the loss of a sense, but with a shift in preferred input sensory modality. According to the theory, the relative weights of different sensory input modalities (e.g., vision, touch, hearing) within an area are related to how useful the information in that modality is for the area’s core function (e.g. motion detection, object recognition etc). Information from the different areas is presumably integrated and coordinated by some kind of ensemble mechanisms, especially during periods of adjustment after the loss of a sensory modality (e.g. through blindness) [6]. Indeed, these kinds of observations have led to an argument that ensembles of neurons, rather than single neurons, should be viewed as the basic functional unit of the central nervous system [12–15].

The examples above are reminiscent of the kinds of effects seen in both cooperative and competitive ensemble methods known to be effective in machine learning [16–20]. Hence a number of researchers have implemented ensemble models that attempt to reflect aspects of the biology while borrowing ideas and methods from machine learning. These include low-level models concentrating on the oscillatory properties of neuron ensembles, showing how synchronisation dynamics between ensembles can underpin supervised and unsupervised adaptation in a variety of scenarios [14, 21–23], and higher-level models proposing information processing architectures that can be used to coordinate and organise learning in ensembles in the brain [5, 6]. In the latter category, mixture of experts (MoE) type architectures [24] have been proposed as an interesting candidate for ensemble learning in the cortex and other areas. In particular Bock and Fine (2014) [6] have argued that a MoE architecture is a very good fit to the functional constancy theory of cortical operation.

**Fig 1:**
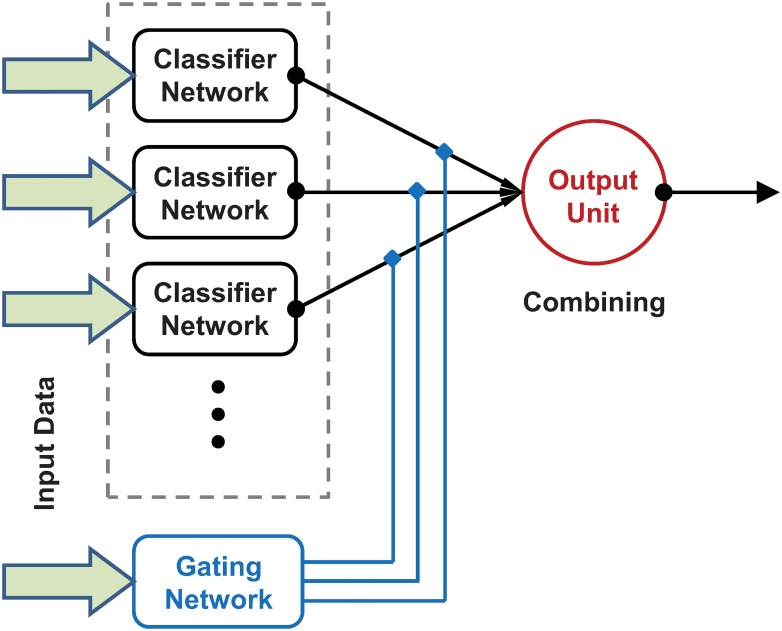
The standard MoE architecture. The outputs (classifications) from the classifier networks are fed into an output unit which combines them according to some simple rule. The gating network weights the individual classifier outputs before they enter the final output unit, and thus guides learning of the overall combined classification. The classifiers and gating networks receive the same input data. See text for further details.

In the artificial neural network literature, ensemble learning on a classification task typically involves multiple continuous value (i.e. on-spiking) artificial neural networks (classifiers) acting in parallel on the same stimuli (pattern to classify), or on different aspects, or modes, of the same overall stimuli. A combined classification from the multiple classifiers, e.g. by majority vote, very often gives better, more reliable performance than that of a single classifier [18, 20]. The MoE ensemble learning architecture makes use of the input stimuli not only to train the individual classifiers (experts) but also to control the mechanism that combines the outputs of the individual experts into an overall classification. In the classic MoE architecture [24], the individual classification outputs of the experts are non-linearly combined via a single gating network which also receives the same input stimuli as the experts (Fig 1). One of the attractions of this architecture is its tendency to cluster input-output patterns into natural groupings, such that each expert can concentrate on a different sub-region of input space (or a different set of sub-problems or ‘tasks’). The gating network tends to guide adaptation in the individual classifiers such that the task space is divided up so as to reduce interference.

The suggestions of MoE type architectures at play in the brain are intriguing but to date there have been no detailed, implementation-level, proposals for biologically plausible, unsupervised, spike-based architectures that exhibits such ensemble learning effects. In this paper, for the first time, we put forward a detailed hypothesis of how experimentally observed neural mechanisms of plasticity can be combined to give an effective and biologically plausible ensemble learning architecture. We demonstrate such an architecture through the computational implementation of a model of unsupervised learning in an ensemble of spiking networks.

One key problem to overcome was how the outputs of multiple networks/areas/‘experts’ could be combined via a non-linear gating mechanism in a biologically plausible way. We propose that a mechanism based on input timing dependent plasticity (ITDP) provides a solution. ITDP, a form of heterosynaptic plasticity activated by correlations between different presynaptic pathways [25, 26], is a rather understudied mechanisms of plasticity but it has been shown to occur in the cortex [27], the cortico-amygdala regions [28] involved in the odour discrimination task mentioned earlier [3], as well as in the hippocampus [26]. We argue that it is a good candidate for the kind of coordination needed in biological ensemble learning mechanisms, particularly as it has recently been shown to involve exactly the kind of gating plasticity mechanisms that would be required in our hypothesized architecture [29].

Nessler et al. (2013) [30] recently proposed a spiking model of cortical microcircuits that are able to perform Bayesian inference. They model the soft winner-take-all (WTA) circuits, involving pyramidal neurons inhibiting each other via interneurons, which have been shown to be a common motif of cortical microcircuits [31]. A combination of spike timing dependent plasticity (STDP) and activity-dependent changes in the excitability of neurons is able to induce Bayesian information processing in these circuits such that they are able to perform expectation maximisation (EM). The circuits are thus referred to as SEM networks (spiking EM) [30]. Our ensemble architecture makes use of such SEM networks as the individual ensemble units (classifiers).

### Mixture of Experts

The standard MoE architecture [24, 32] used in machine learning is shown in Fig 1. The outputs of an ensemble of *N* classifiers feed into a final decision unit whose output is the combined classification. A separate gating network, with *N* outputs, weights the individual classifier outputs, typically by multiplying them by the corresponding gating output (Fig 1). The final decision unit uses a simple rule (often some variation of the highest weighted classification from the ensemble classifiers) to generate the final classification. The classifiers and the gating network are typically feedforward nets which are trained by a gradient descent algorithm in a supervised manner. In the standard setup the classifiers in the ensemble and the gating network all receive the same input data. The classifiers and the combining mechanism, via the gating network, adapt together, with the gating mechanism helping to ‘guide’ learning. This often leads to some degree of specialization among the ensemble with different classifiers performing better in different areas of the input space. Extensions can include more explicit variation among the classifiers by providing them with different inputs (e.g. different sub samples, or features, of some overall input vector). Techniques such as this can encourage diversity among the classifiers which is generally a good thing in terms of performance [18]. In general, ensemble methods, such as MoE, have been shown to outperform single classifier methods in many circumstances. The combined performance of an ensemble of relatively simple, cheap classifiers is often much better than that of the individual classifiers themselves [16, 18, 20].

Our model of ensemble learning in biologically plausible spiking neural networks does not attempt to slavishly follow the methods and structure of the standard MoE architecture, but instead adapts some of the basic underlying principles to produce a MoE like system which can operate according to biologically plausible mechanisms which are based on empirical findings.

### Input Timing Dependent Synaptic Plasticity

**Fig 2:**
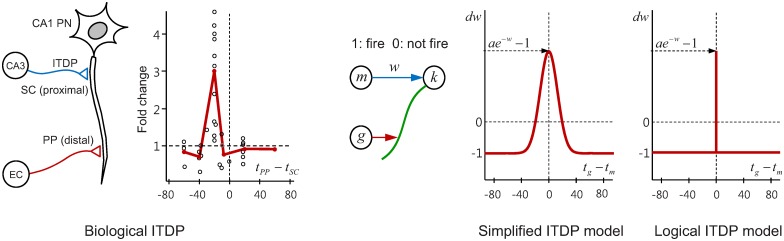
Experimentally observed ITDP behaviour (left) (after [26]), and its simplifications (right) used in this paper. The original ITDP behaviour is modelled either by a Gaussian (for spiking neural network) or a pulse (for logical voter network) functions.

The term input timing dependent plasticity (ITDP) was first coined in [26] where it was empirically demonstrated in the hippocampus. It is a form of heterosynaptic plasticity—where the activity of a particular neuron leads to changes in the strength of synaptic connections between *another* pair of neurons, rather than its own connections. Classical Hebbian plasticity involves correlations between pre- and post- synaptic activity, specifically activity in the presynaptic cell is causally related to activity in the postsynaptic cell [33]. By contrast, ITDP involves synaptic plasticity which is induced by correlations between two presynaptic pathways. Dudman et al. (2007) [26] observed that stimulation of distal perforant path (PP) inputs to hippocampal CA1 pyramidal neurons induced long-term potentiation at the CA1 proximal Schaffer collateral (SC) synapses when the two inputs were paired at a precise interval. The neural system is illustrated in Fig 2 left. Plasticity at the synapse (SC) between neurons CA3 and CA1 is induced when there is a precise interval between stimulations from CA3 and from the distal (PP) perforant pathway from neuron EC in the entorhinal cortex (see timing curve, Fig 2 left). More recently, Basu et al. (2016) [29] have extended these findings by investigating the role of additional long-range inhibitory projections (LRIPs) from EC to CA1, the function of which were largely unknown. They showed that the LRIPs have a powerful gating role, by disinhibiting intrahippocampal information flow. This enables the induction of plasticity when cortical and hippocampal inputs arrive at CA1 pyramidal neurons with a precise 20ms interval.

Humeau et al. (2003) [25] observed a very similar form of heterosynaptic plasticity in the mammalian lateral amygdala. Specifically, simultaneous activation of converging cortical and thalamic afferents induced plasticity. More recently ITDP has been demonstrated in the cortex [27] and in the cortico-amygdala regions [28]. Another study [34] predicted the function of the vestibule-occular reflex gain adaptation by modeling heterosynaptic spike-timing dependent depression from the interaction between vestibular and floccular inputs converging on the medial vestibular nucleus in the cerebellum. Dong et al. (2008) [35] also reported a related kind of heterosynaptic plasticity operating in the hippocampus, but on different pathways from those studied by Dudman et al (2007) [26] and Basu et al. (2016) [29]. Thus this, as yet little studied, form of plasticity appears to exist in many of the main brain regions associated with learning and the coordination of information from multiple sensory/internal pathways.

In the above example of ITDP acting in the hippocampus (Fig 2), the role of neuron EC in enabling ITDP driven plasticity at synapse SC is somewhat reminiscent of the action of the gating neurons in the MoE architecture outlined in the previous section, especially when we take into account the new findings that the EC to CA1 inhibitory projections do indeed enable a gating mechanism [29]. Moreover, distal projection from the entorhinal cortex to the CA1 region are topographic [36, 37] and the enhancement of excitatory postsynaptic potentials (EPSP) is specific to the paired pathway [26], indicating that only the ITDP synapse which is paired with the distal signal is potentiated. These facts suggest the possibility of specific targeted pathways enabling ‘instructor’ signals. In addition, the EPSP from the distal input is attenuated [26], meaning that the ‘instructor’ signal would not directly influence any final network output, rather it indirectly influences through ‘instructions’ that enable plasticity. These properties are exactly those needed to operate a biologically plausible spiking MoE type architecture. This led us to the development of such an architecture using an ensemble of spiking networks with ITDP-activating distal connections playing a kind of gating role which allows coordinated learning in the ensemble (these connections are a slight abstraction of the PP and LRIP connections rolled into one, to provide a temporally precise mechanism). This system is described over the following sections and embodies our biologically founded hypothesis of a potential role for ITDP in coordinating ensemble learning.

First a tractable analytic model of the biologically plausible ITDP driven spiking ensemble architecture and its attendant MoE type mechanisms is developed. Derived from a logical model based on the probabilities of neural firing events, this gives insights into the system’s performance and stability. With this knowledge in hand, the analytic model is extended into a full, biologically plausible, computational implementation of the architecture which is demonstrated on a visual classification task (identifying hand written characters). The unsupervised ensemble learning mechanism is shown to perform the task well, with the combined ensemble performance being significantly better than that of the individual classifiers. The properties of the full model are analysed in the light of extensive experiments with the classification task, including an investigation into the influence of different input feature selection schemes and a comparison with a hierarchical STDP-only based ensemble architecture.

## Results

### An Analytic Model of a Voter Ensemble Network with ITDP

This section describes the analytic formulation of ITDP driven spiking ensemble learning using probability metrics. The development of such an analytic/logical model serves two purposes: to demonstrate and better understand the mechanisms of spike-based ensemble learning, particularly the coordination of classifier outputs through ITDP, and as the basis of a fast, simplified model which can be used to provide unsupervised learning in an ensemble of arbitrary base classifiers. Later in the paper we extend the proposed model to a more biologically plausible spiking neural network ensemble learning architecture.

#### Three neuron ITDP

We developed a tractable model based on the hippocampal system in which Dudman et al. (2007) [26] first demonstrated ITDP empirically. Consider three simplified binary ‘neurons’ which ‘fire’ an event (spike) according to their firing probabilities. The first neuron *k* represents a target neuron which corresponds to the hippocampal CA1 pyramidal cell (Fig 2), the second neuron *m* represents a CA3 neuron which projects a fast Schaffer collateral (SC) synapse to the proximal dendrite of *k*, and the last neuron *g* represents a neuron from the entorhinal cortex that projects a distal (PP) synapse via a perforant pathway to the CA1 cell. *g* is modelled as a gating neuron.

For analytical tractability, we first consider a discrete-time based system as an extremely simplified case. We assume output of the system is clocked, where all neurons always give their decisions synchronously by either firing or being silent at every tick. The distal firing delay (20ms) of biological ITDP is eliminated by ignoring the effects of hippocampal trisynaptic transmission delay and the deformation of distal excitatory postsynaptic potentials (EPSPs) due to dendritic propagation. Thus the potentiation of the ITDP synapse occurs only when the two presynaptic neurons fire together at any given time instance. This plasticity rule can be conceptually illustrated by simplifying the original experimental ITDP curve as a pulse-like function (Logical ITDP model in Fig 2), where we can regard the ITDP operation as a logical process which is modelled as “(*m*, *g*) fire together, (*m*, *k*) wire together” in a heterosynaptic way. A model using a Gaussian simplification which takes the proximal-distal spike interval into account (Simplified ITDP model in Fig 2) will be used later for a more detailed, biologically plausible neural network model, where each presynaptic neuron fire a burst of spikes as an output event thus having a range of different spike-timings between two presynaptic neurons. For the time being we concentrate on the logical model which allows us to examine some important intrinsic properties of learning in a spiking ensemble. From this logical simplification, we can express the probabilities of the possible joint events of two presynaptic neurons with independent Bernoulli random variables *m* and *g* at any discrete time instance as:

$$\begin{array}{c} p(m\wedge g)=p(m)p(g) \end{array}$$ (1)

$$\begin{array}{c} p(m\vee g)=p(m)+p(g)-p(m)p(g) \end{array}$$ (2)

$$\begin{array}{c} p(\neg m\wedge \neg g)=1-p(m\vee g) \end{array}$$ (3)

We assume *m* and *g* to be independent in this simplified illustrative model in line with the (hippocampal) biological case where the input signals for neurons *m* and *g* are assumed to be uncorrelated. This is because whereas *g* receives direct sensory information from EC, *m* receives highly processed information of the same sensory signal through a tri-synaptic path, so the inputs for the two neurons can essentially be assumed to be independent. In the full ensemble models developed later, this assumption holds, to a good level of approximation, as the input vectors for each ensemble classifier are distinct measurements of the raw input data through the use of different feature subsets for each classifier. This issue is discussed further in Methods.

The synaptic weight *w* in this logical model is potentiated by ITDP when both *m* and *g* fire. In order to prevent the unbounded growth of weight strength, we employed the synaptic learning rule from [30], such that the synapse is potentiated by an amount which is inversely exponentially dependant on its weight, whereas it is depressed by a constant amount if only one neuron *m* or *g* fires. If neither of the presynaptic neurons fire, no ITDP is triggered. This self-dependent rule is not intended to model the slight amount of LTD which was originally shown in the outer region of the peak potentiation of the experimental ITDP curve shown by [26] (see Fig 2 left). Rather, it provides a local mechanism for synaptic normalisation where multiple proximal synapses from a number of *m* neurons compete for the synaptic resources without the unbounded growth of synaptic weights. Also it has been shown that the kind of inversely exponential weight dependency rule used here closely reproduced the pre-post pairing frequency dependent STDP behaviour of biological synapses [30] when used to model STDP. It is expected that this correspondence will also be valid for other types of timing-dependent plasticities such as ITDP. Thus, using this rule, the weight change by ITDP in our logical model is triggered when either one of *m* or *g* or both fire. The change of the weight Δ*w* from neuron *m* to the postsynaptic neuron *f* can be written as:

$$\Delta w\;=\{\begin{array}{ll} ae^{-w}-1\;, & \text{if }(m\wedge g)\\ -1\;, & \text{if }(m\vee g)\wedge \neg (m\wedge g)\\ 0\;, & \text{otherwise } \end{array}$$ (4)

where *a* ≥ 1 is a constant which shifts the weight to a positive value. It is evident that the sum of all three probabilities is 1 according to Eqs 1–3. From Eqs 1–4, we derived the expected value of the weight *w* at equilibrium under constant presynaptic firing probabilities to give the expression in Eq 5 (see Methods for details).

$$\begin{array}{c} {w|}_{\text{E}[\Delta w]=0}=\text{log}\left(a\right)+\text{log}\left(\frac{p(m)p(g)}{p(m)+p(g)-p(m)p(g)}\right) \end{array}$$ (5)

Now we have the expected value of *w* at equilibrium expressed in terms of the two probabilities *p*(*m*) and *p*(*g*). It can be seen that the weight converges to the difference of two log probabilities of the events (*m* = 1 and *g* = 1) and (*m* = 1 or *g* = 1) with a shift of log(*a*).

### Unsupervised Learning in a Spiking Voter Ensemble Network

**Fig 3:**
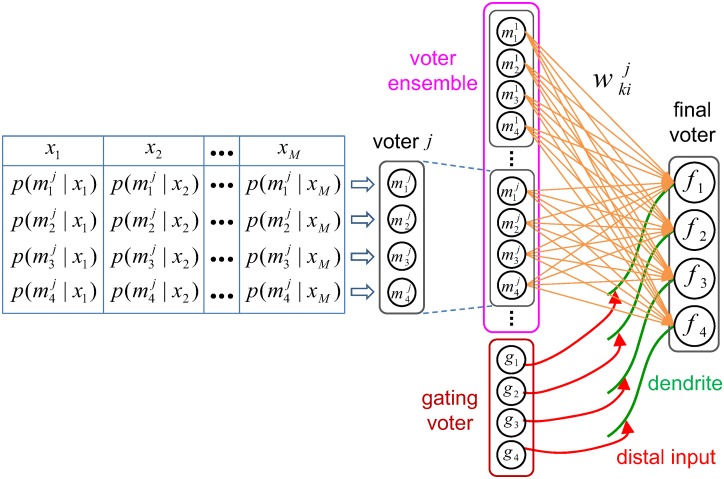
A voter and the voter ensemble network (*N*_*C*_ = 4). (Left) A voter and the predefined firing probabilities of each voter neuron for a set of virtual input samples *X* = {*x*_1_, *x*_2_, …, *x*_*M*_}. (Right) The voter ensemble network. The weight $w_{ki}^{j}$ represents the weight of connection from the *i*th neuron of the *j*th voter to the *k*th neuron of the final voter.

Next we built an extended logical model for learning the weighted combination of a population (ensemble) of spiking neuronal voters (classifiers) using the simplified ITDP model described earlier. A voter was assumed to have a set of output neurons (one for each class) each of which fires an event (spike) according to its firing probability distribution. The voter follows the mechanism of stochastic winner-takes-all (sWTA), where only a single neuron can fire for any presented input data. The firing probabilities of the neurons in a voter sum up to unity and these probabilities are determined by the input presented to the voter. Therefore, a voter generates a stochastic decision (casts a vote representing the classification) by firing a spike from one of its output neurons whenever an input pattern is presented to the voter. The input pattern shown to the voter can be any neurally coded information (such as an image, sound, or tactile information) which is to be classified by the voter. A pattern given to the voter is regarded as being labeled as belonging to a certain class (c), where the number of existing classes is assumed to be initially known. However, it is unnecessary to relate the absolute value of the class label to the specific neuron index, since any voter neuron can represent an arbitrary data class by firing dominantly. In this abstract model, which was primarily motivated as a vehicle to test the efficacy of ITDP driven coordination of ensemble member outputs, the individual ensemble classifiers were assumed to be fully trained in advance using an arbitrary set of input data. Their tables of firing probabilities (as in Fig 3) effectively represent the posterior probabilities of each class for a given input vector.

Using the simplified voter model, we can build an analytically tractable voter ensemble network capable of learning the spike-based weighted combination of the individual voters. In other words, learn to combine the individual votes by weighting them appropriately so as to give a better overall classification. The ensemble system consists of three subsystems similar to those in the MoE architecture: an ensemble of voters, a final voter which receives the decisions from the ensemble and combines them to give the final classification output, and a gating voter which guides ITDP between the ensemble and the final voter (Fig 3 right). The neurons of all voters in the ensemble project connections to all the neurons in the final voter (c.f. proximal projections from CA3 in the hippocampal case), whereas the gating voter projects topographic (one to one) distal connections to the final voter (Fig 3 right, c.f. distal topographic projections from EC in the hippocampal case). Every ensemble voter and the gating voter take their own representation vectors derived either from the same input pattern or from different patterns from distinct input subsets (e.g. different regions of an image). The spikes from the gating voter passing through the topographic distal connection are assumed to have no significant contribution to the final voter output (except indirectly through guiding ITDP). This is because, following the biological data, in our model long range EPSP propagation from distal synapses to the soma is significantly attenuated and therefore has little influence on evoking postsynaptic action potentials [26].

The gating voter guides ITDP via its topographic projections, which selectively enhance the connection strengths from the ensemble voter neurons representing the same class to one of the final voter neurons (the gating voter’s topographic counterpart) regardless of the ensemble neuron indices. Therefore, the system produces the ‘unsupervised’ weighted combination of ensemble outputs by learning the ITDP weights to reflect the long term co-firing statistics of the ensemble and the gating voter so that the most coherent neuronal paths for a specific class are converged to one of the final voter neurons.

We derived the following analytic solution (Eq 6) for the values of the weights of the ITDP synapses projecting from the voter ensemble to the final voter (Fig 3) under equilibrium (i.e. when they have converged after learning). See Methods for details of the derivation.

$$\begin{array}{c} w_{ki}^{j}=\text{log}\left(a\right)-\text{log}\left(\frac{\sum_{l=1}^{M}\left\{p\left(m_{i}^{j}|x_{l}\right)+p\left(g_{k}|x_{l}\right)\right\}}{\sum_{l=1}^{M}p\left(m_{i}^{j}|x_{l}\right)p\left(g_{k}|x_{l}\right)}-1\right) \end{array}$$ (6)

Where $p(m_{i}^{j}|x_{l})$ is the firing probability of the *i*th neuron of the *j*th ensemble voter for input sample *x*_*l*_, $w_{ki}^{j}$ is the weight from $m_{i}^{j}$ to the *k*th neuron (*f*_*k*_) of the final voter, and *p*(*g*_*k*_|*x*_*l*_) is the firing probability of the corresponding gating voter neuron which projects to *f*_*k*_.

We also derived an analytic solution for the expected firing probability of a final voter neuron under the presentation of the samples belonging to a particular class as given in Eq 7 (see Methods for derivation).

$$\begin{array}{c} \text{E}\left[p\left(f_{k}|c\right)\right]=\frac{1}{M_{c}}\sum_{l=1}^{M_{c}}\sum_{q=1}^{N_{S}}\left(p\left(s_{q}|x_{l}\right)\cdot \frac{e^{u_{k}\left(q\right)}}{\sum_{r=1}^{N_{C}}e^{u_{r}\left(q\right)}}\right), \end{array}$$ (7)

where *p*(*f*_*k*_|*c*) is the firing probability of a final voter neuron at *q*th ensemble state *s*_*q*_ under presentation of the samples from class *c*, *u*_*k*_(*q*) is the weighted sum of spikes from the ensemble in state *s*_*q*_ arriving at the postsynaptic neuron *k*, and *N*_*C*_ is the number of classes (see Methods for full explanation of all terms). This gives the analytic solution of the final voter firing probabilities as a function of joint probabilities of ensemble voter firings under each class presentation. The addition of these expression now gives us a complete analytic spiking ensemble model.

#### Validation of analytic solutions by numerical simulation

In order to see if the ensemble architecture performs as expected and to validate the analytic solutions of the voter ensemble network, we compared its results, as derived in the previous section, with a numerical simulation that simply iterated through all the underlying equations of the same model. This validation was deemed worthwhile because the simplified analytical model is based on Bernoulli random variables that simulate per sample firing events. The numerical simulation of the model allowed us to check that the long-term trends and statistics matched those predicted by the analytical solutions. Full details can be found in S1 Text.

The simple iterative numerical simulation—using abstract input data—did indeed produce very close agreement with the analytic solutions, validating our analytic formulation of expected weight values, and demonstrated that the system performs very well under appropriate parameter settings. By defining a number of parameters that easily allowed us to design a range of differently performing ensembles, the simple numerical simulation also allowed various insights into the overall learning dynamics and the dependence on key factors (ensemble size, gating voter performance, ensemble voter performances). The performance of classifiers (voters) was measured using normalised conditional entropy (NCE) [30], which is suitable for measuring the performance of a multi-class discrimination task where the explicit relation between the neuronal index and the corresponding class is unavailable. NCE has a value in the range 0 ≤ NCE ≤ 0.5, with lower conditional entropy indicating that each neuron fires more predominantly for one class, hence giving better performance—this measure will be used throughout the remainder of this paper (see Methods for the details of the simulation procedure and the NCE calculation, see S1 Text for full details of the simple numerical simulation results).

One key insight confirmed by the simple numerical simulation was that, as long as there is sufficient guidance from the gating voter, the decisions from the better performing ensemble neurons influence the final voter output more by developing relatively stronger weights than the other neurons. Thus the spike from one strongly weighted synaptic projection can overwhelm several other weakly weighted ‘wrong’ decisions. Such dynamics achieved successful learning of the weighted vote, based on the history of ensemble behaviour (exactly the behaviour we desire in this kind of ensemble learning). More specifically, the simulation of the simplified spiking ensemble system showed that the gating voter and at least one ensemble voter must have positive discriminability (NCE<0.5) in order to properly learn to perform weighted voting. That is, the gating voter, and at least one ensemble member, must have at least reasonable—but not necessarily great—performance on the classification task for the overall ensemble performance to be very good.

These validation tests showed that the logical model of a spiking voter ensemble system and its analytic solutions are capable of performing efficient spike-based weighted voting, driven by ITDP, and gave us important insights into how that is achieved. They also demonstrated how the seemingly complex network of interactions between stochastic processes within a population of voters can be effectively described by a series of probability metrics. In the next section we report on results from a computational model based on this tractable logical model which was significantly extended to encompass more biologically realistic spiking neural networks, with ensemble members having their own inherent plasticity. This system was demonstrated on a practical classification task with real data.

### Ensemble of ITDP Mediated Spiking Expectation Maximization Neural Networks

The logical voter ensemble model described in the previous section showed that the computational characteristics of ITDP provide a novel functionality which can be used to coordinate multiple neural classifiers such that they perform spike based online ensemble learning. This form of ensemble learning simultaneously solves both the weighted vote and combining problems of arbitrarily ordered decisions from individual classifiers in an unsupervised manner. After this validation of the overall ensemble scheme, we next investigated an extended neural architecture for combined learning in an ensemble of biologically plausible spiking neural network classifiers using ITDP. The overall scheme is based on the initial simplified model, but the components are now significantly extended. Instead of assuming the individual classifiers are pre-trained, they are fully implemented as spiking networks with their own inherent plasticity. Individual classifier and overall ensemble learning dynamics occur simultaneously. The individual classifiers in the ensemble are implemented as Spiking Expectation Maximisation (SEM) neural network which have been shown to perform spike based Bayesian inference [30], an ability that is often cited as an important mechanism for perception [38–40] in which hidden causes (e.g. the categories of objects) underlying noisy and potentially ambiguous sensory inputs have to be inferred.

A body of experimental data proposes that the brain can be viewed as using principles of Bayesian inference for processing sensory information in order to solve cognitive tasks such as reasoning and for producing adequate sensorimotor responses [41, 42]. Learning using Bayesian inference updates the probability estimate for a hypothesis (a posterior probability distribution for hidden causes) as additional evidence is acquired. Recently, a spike-based neuronal implementation of Bayesian processing has been proposed by Nessler et al. [30, 43, 44] as a model of common cortical microcircuits. Their feedforward network architecture implements Bayesian computations using population-coded input neurons and a soft winner takes all (WTA) output layer, in which internal generative models are represented implicitly through the synaptic weights to be learnt, and the inference for the probability of hidden causes is carried out by integrating such weighted inputs and competing for firing in a WTA circuit. The synaptic learning uses a spike-timing dependent plasticity (STDP) rule which has been shown to effectively implement Maximum Likelihood Estimation (MLE) allowing the network to emulate the Expectation Maximization (EM) algorithm. The behaviour of such networks was validated by a rigorous mathematical formulation which explains its relation to the EM algorithm [30].

**Fig 4:**
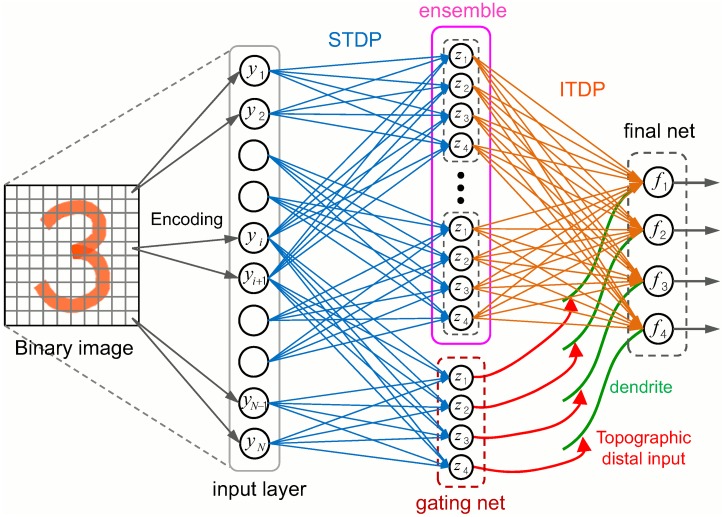
SEM-ITDP ensemble network architecture. The STDP connections, which projects from the selected input neurons to each WTA circuit, together with the WTA circuits constitute the SEM ensemble. The ITDP connections have the same connectivity as the logical ITDP model. All of the ensemble, gating and final output networks use the same SEM circuit model.

Our reimplementation and extension of Nessler’s [30] model forms the basis of our classifiers and is well-suited for integration into our spike-based ensemble system. Viewing the SEM model as a unit cortical microcircuit for solving classification tasks, we can naturally build an integrated ITDP-based ensemble architecture as an extension of the logical ITDP ensemble model described earlier. Fig 4 shows the two layer feedforward neural architecture for the SEM-ITDP ensemble system. The first layer consists of an ensemble of SEM networks and a gating SEM, which share the presynaptic input neurons encoding the input data. Reflecting the often non-uniform, and specifically targeted, convergent receptive fields of cortical neurons involved in perceptual processing [45], each WTA circuit receives a projection from a subset of input neurons (representing e.g. a specific retinal area), which enables learning for different ‘feature’ subsets of the input data. All synapses in the ensemble layer are subjected to STDP learning. Following Nessler et al. (2013) [30] and others, in order to demonstrate and test the operation of the system, binarized MNIST handwritten digit images [46] were used as input data for classification, where the ON/OFF state of each pixel is encoded by two input neurons. The MNIST dataset is a large database of handwritten digits covering a wide range of writing styles, making it a challenging problem. The output from the ensemble layer is fed to the final WTA circuit via ITDP synapses which are driven by the more biologically plausible ITDP curve shown in Fig 2. The following sections will describe in detail the model SEM circuit and the ITDP dynamics, followed by an investigation into how the SEM-ITDP ensemble system applied to image classification performed simultaneous realtime learning of both the individual classifier networks and the ITDP layer in parallel.

#### SEM neural network model

Let us first revisit a single SEM neural network model [30] for spike based unsupervised classification. The SEM network is a single layer spiking neural network in which the neurons in the output layer receive all-to-all connections projected from a set of inputs. The output neurons are grouped as a WTA circuit which is subjected to lateral inhibition, modelled as a common (global) inhibitory signal which is in turn based on the activity of the neurons. A WTA circuit consists of *K* stochastically firing neurons. The firing of each neuron *z*_*k*_ is modelled as an inhomogeneous Poisson process with instantaneous firing rate *r*_*k*_(*t*),

$$\begin{array}{c} r_{k}\left(t\right)=e^{u_{k}\left(t\right)} \end{array}$$ (8)

$$\begin{array}{c} u_{k}\left(t\right)=w_{k0}+\sum_{i=1}^{n}w_{ki}y_{i}\left(t\right)-I\left(t\right)+v\left(t\right) \end{array}$$ (9)

$$\begin{array}{c} I(t)=O_{\text{inh}}-\left(A_{\text{inh}}+O_{\text{inh}}\right)e^{\left(t_{f}-t\right)/\tau_{\text{inh}}} \end{array}$$ (10)

where *u*_*k*_(*t*) is a membrane potential which sums up the EPSPs from all presynaptic input neurons (*y*_*i*_ (*i* = 1, …, *n*)) multiplied by the respective synaptic weight *w*_*ki*_. The variable *w*_*k*0_ represents neuronal excitability, and *I*(*t*) is the input from the global inhibitory signal to the WTA circuit. *v*(*t*) is an additional stochastic perturbation by a Ornstein-Uhlenbeck process which emulates the background neural activity using a kind of simulated Brownian dynamics that decorrelates the WTA firing rate from that of the input firing rate in order to prevent mislearning [30, 47]. The EPSP evoked by the *i*th input neuron is modelled as a double exponential curve which has both fast rising (*τ*_*f*_) and slow decaying (*τ*_*s*_) time constants. At each time instance, EPSP amplitudes are summed over all presynaptic spike times (*t*_*p*_) to become *y*_*i*_(*t*) for the *i*th input at time *t*.

$$\begin{array}{c} y_{i}\left(t\right)=A_{\text{EPSP}}\sum_{t_{p}}\left(e^{\frac{t_{p}-t}{\tau_{s}}}-e^{\frac{t_{p}-t}{\tau_{f}}}\right) \end{array}$$ (11)

The scaling factor *A*_EPSP_ is set as a function of the two time constants in order to ensure that the peak value of an EPSP is 1. Whenever one of the neurons in the WTA circuit fires at *t*_*f*_, *I*(*t*) adds a strong negative pulse (amplitude of *A*_inh_) to the membrane potential of all *z* neurons, which exponentially decays back to its resting value (*O*_inh_) with a time constant (*τ*_inh_). Therefore, *I*(*t*) determines the overall firing rate of WTA circuits as well as controlling the refractory period of a fired neuron.

Input evidence *x*_*j*_ for a feature *j* of observed data is encoded as a group of neuronal activations *y*_*i*_. If the set of possible values of *x*_*j*_ consists of *m* values *G*_*j*_ = [*v*_1_, *v*_2_, …, *v*_*m*_], the input *x*_*j*_ is encoded using *m* input neurons. Therefore, if input data is given as a *N* (*j* = 1, …, *N*) dimensional vector, the total number of input neurons is *mN*. For further details of the Bayesian processing dynamics of the SEM networks see the Methods section.

The rules for STDP driven synapse plasticity between the input layer and the SEM classifiers, ITDP driven plasticity on final output network synapses (as in Fig 4), and neuronal excitability plasticity, are all explained in the Methods section. In this extended version of the model, ITDP follows the biologically realistic plasticity curve shown in Fig 2 middle (Simplified ITDP curve).

#### Experiments with ensembles of SEM networks

In this section we present results from running the full biologically plausible SEM ensemble architecture on a real visual classification task (as depicted in Fig 4). We show that the ensemble learning architecture successfully performed the task and operated as expected from the earlier experiments with the more abstract logical ensemble model (on which it is based). Weights in the STDP and ITDP connection layers smoothly converged to allow robust and accurate classification. The overall ensemble performance was significantly better than the individual SEM classifier performances. The initial experiments used a random (input) feature selection scheme.

The SEM ensemble architecture was tested on an unsupervised classification task involving recognizing MNIST handwritten digits [46]. Each piece of input data was a greyscale image having 28×28 = 784 pixels. The class labels of all data were unknown to the ensemble system, so both the learning and combining aspects of the ensemble are unsupervised. All images were binarized by setting all pixels with intensity greater than 200 (max 255) to 1, and 0 otherwise. The dimension of the binary image was reduced by abandoning less occupied pixels by preprocessing over the entire images in the dataset (pixels being ‘on’ in less than 3% of the total image presentation were disabled) [30].

In contrast to the logical voter model experiments, where output was manually designed to produce stochastic decisions, the outputs of individual SEM networks using a real dataset tend to produce the same decision error for the specific input data. Promoting diversity between individual classifier outputs is a prerequisite for improving ensemble quality in the machine learning literature [48, 49], and ensemble feature selection has been shown to be an essential step for constructing an ensemble of accurate and diverse base classifiers. The features of an image in biological visual processing generally implies the neurally extracted stimuli which represent the elementary visual information of a scene (such as spot lights, oriented bars, and colors), and they need to be learnt through the layers of a neural pipeline [50–52] which is beyond the scope of this work. For the sake of simplicity, we used a raw pixel as the basic feature which could be selected as an informative subset of the input data space. It has been shown that specific forms of weight decay or regularization provide a mechanism for biologically plausible Bayesian feature selection [53–55]. In our ensemble system, selective projections from the input layer to the ensemble WTAs effectively implemented pixel/feature selection in this regard. Each ensemble layer SEM network learnt over a distinct subregion of images by neurally implementing ensemble feature selection, where each ensemble WTA circuit received the projection from a selected subset of input neurons such that the all-to-all connectivity from a pair of input neurons *m* and *m* + 1 to the WTA neurons was enabled if the pixel *m* was selected as a feature. A quarter of the total number of pixels were selected for each ensemble member by the feature selection schemes used (described later).

The gating network used either full (i.e. the whole image) or partial features for testing supervised or unsupervised gating of ITDP learning. In order for both the partial-featured ensemble network and the full-featured gating network to receive input from the same number of input neurons, the images were supersampled to 56×56 pixels. This is because the output of our WTA circuit is a train of spikes (typically bursting at a few tens of Hz) during input presentation, and different numbers of input neurons may result in different numbers of spikes in an output burst. For ITDP learning, it is logically compatible with biological ITDP *in vitro* (both distal and proximal neurons fire a single spike) to make all the ensemble WTAs and the gating WTA fire the same number of spikes per burst. The image supersampling replicated a pixel to four identical pixels (all four pixels indicate the same feature), so the set of all features for the gating WTA was represented by a quarter of the pixels of the supersampled image. A quarter of pixels were selected for each ensemble WTA as its feature subset using some selection scheme (see later). Thus the same number of input pixels was achieved both for the ensemble and gating WTAs. Another way of thinking about this process is that the pixels selected for an ensemble WTA were replicated in order to match their number to the size of the original (not supersampled) image.

We conducted an initial experiment using four classes of images (digits 0, 1, 2, and 3) each of which had 700 samples (2800 images in total). The original 784 pixels were reduced to 347 by dimensionality reduction, followed by supersampling them to *m* = 1388, hence there were *N*_*I*_ = 2*m* = 2776 input neurons in the input layer and *K* = 4 output neurons in each WTA circuit. The number of synapses is proportional to *N*_*E*_ as each ensemble WTA receive the same number of inputs in order to give an output burst of regular numbered spikes which behaves as similar as possible to the (earlier tractable) logical voter ensemble model (which had been shown to perform well). Given an ensemble size *N*_*E*_, the system has *KN*_*I*_(*N*_*E*_ + 1)/4 STDP synapses in the first layer, *K*^2^ *N*_*E*_ ITDP synapses in the second layer, and *N*_*I*_ + *K*(*N*_*E*_ + 2) Poissonian neurons. The effect of increased synapses in the second layer was compensated by adjusting the inhibition level of the final WTA circuit (See Methods). We initially used random feature selection, where a quarter of pixels were randomly selected for each ensemble member and for the gating network, and the corresponding input neurons projected STDP synapses to their target WTA circuit. The input was fed to the network by successively presenting an image from one class for a certain duration (*T*_*present*_), followed by a resting period (*T*_*rest*_) where none of the input neurons fire, in order to avoid overlap of EPSPs from input spikes caused by different input images. Full numerical details of the experimental setting can be found in Methods (subsection SEM-ITDP experiments).

**Fig 5:**
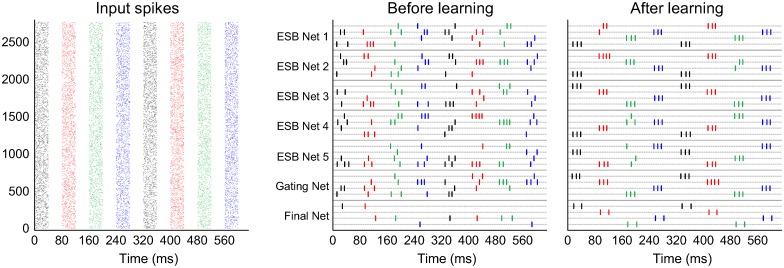
Spike trains from the SEM ensemble network with *N*_*E*_ = 5 and random feature selection. (Left) Plot shows the input neuron spikes from eight image presentations from different classes (digits) which are depicted in different colors (black: 0, red: 1, green: 2, blue: 4). (Right) Two graphs show the output spikes of ensemble, gating, and final WTA neurons before and after learning. The colors of the spikes represent which class is being presented as input. After learning the network outputs produce consistent firing patterns, each output spiking exclusively for a single class.

An example of the ensemble classification learning task with random feature selection is shown in Fig 5. One of the images in a class was presented for 40ms followed by another 40ms of resting period. Different images generated from four classes were presented successively in a repeating order. Approximately a few tens of seconds after starting the simulation, the output neurons of all WTA circuits began to fire a series of ordered bursts almost exclusively to one of the hidden classes of each presented image. The allocation of output neuron indices firing for a specific class arbitrarily emerged in all of the ensemble layer WTAs by unsupervised learning, whereas the neuron indices between the gating network and final network were matched by ITDP guidance. Technically, the system is not completely unsupervised because the number of classes is provided, even if the class labels are not; however, blinding the class labels makes the task challenging for the system which has to discriminate distinct hidden causes in a self-organised manner. It can be seen from the figure that, after a period of learning, the network outputs produce consistent firing patterns, each output spiking exclusively for a single class of input data.

**Fig 6:**
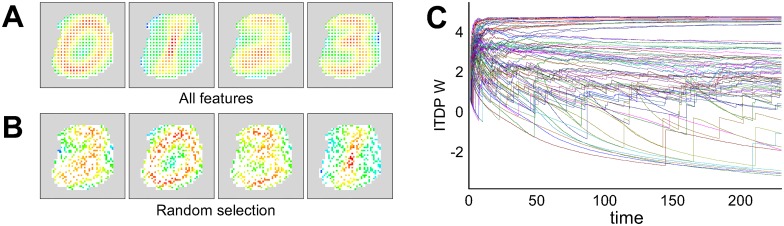
An example of the STDP weight maps of a SEM classifier after learning (A, B) and the time evolution of ITDP weights (C). Each weight map represents the presynaptic weight values that project to each of four WTA neurons (which each fire dominantly for one of the classes). The grey area shows pixels disabled by preprocessing, and each colored pixel represent the difference of the weights from the two input neurons for the corresponding pixel (white pixels represent unselected features). So as to use all features, a quarter of pixels are evenly selected from the supersampled image in order to use all pixels of the original data.

After learning, the presynaptic weight maps for each output neuron of an ensemble layer WTA circuit clearly represent four different hidden causes, which are shown by depicting the difference between ON and OFF weights for each pixel (Fig 6A and 6B). Once one of the WTA neurons fires for one class more than the others, its presynaptic STDP weights are adjusted such that either the ON or OFF weights for corresponding pixels are enhanced by STDP to reflect the target class. Thus the output neuron comes to fire more when an image from the same class is presented again. Fig 6C shows the emergence of typical ITDP guided weight learning on the connections between the ensemble layer and the final net (Fig 4). Over the learning period weight values become segregated into groups which depend on the frequency of the co-firing of the ensemble and the gating neurons. In most cases, the highest-valued group consisted of projections which formed topographical (but not necessarily using the same index) connections between the neurons of each ensemble WTA and the final output WTA neurons, which meant that the connections carrying the signal for the same class were most enhanced and converged to the corresponding final WTA neurons. Therefore it can be seen that the process for combining ensemble outputs, controlled by ITDP learning, functioned similarly to the learning of a spike-based majority vote system where only topographic connections having identical weights exist between each ensemble WTA and the final WTA. Despite the system having no information about the class labels in ensemble WTA neurons, the gating WTA (which is also unsupervised) could selectively recruit and assign the ensemble output to converge to one of the final layer neurons based on the history of the ensemble output. Clearly, the fully extended ensemble architecture performs as expected.

**Fig 7:**
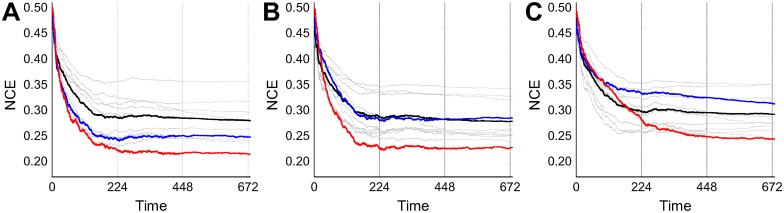
Examples of ensemble behaviours (*N*_*E*_ = 9) for different gating network performances ((A) better than, (B) similar to, (C) worse than the ensemble average). All the ensemble and the gating WTAs used random feature selection. The colors represent the NCEs of the final network (red), the gating network (blue), the ensemble networks (grey) and their average (black). Vertical lines indicate the time span of the total data presentation, where input data are sequentially presented for multiple rounds in order to see long term convergence. The NCE value at time *t* is calculated by counting the class-dependent spikes within the past finite time window of [*T*_*p*_, *t*] (*T*_*p*_ < *t*). In order to prevent a sudden change in the NCE plots due to the exclusion of the early system output (which are immature resulting in high NCE values) from the time window, *T*_*p*_ was dynamically changed for faster burn-out of those initial values as: *T*_*p*_ = *t*(1−*d*/4*D*) where *d* = *t* when *t* < 2*D* and *d* = 2*D* otherwise, *D* = 224sec is the duration of one round of dataset presentation. See Methods for details of the NCE calculations.

The classification performance of the network was represented by calculating the normalised conditional entropy (NCE) as in Eqs 26–28. Low conditional entropy indicates that each output neuron fires predominantly for inputs from one class, hence representing high classification performance. In order to observe the continuous change of network performance over time, the conditional entropy was calculated within a moving time window of 2800 image presentations (the total number of data) which is approximately 224 seconds in simulation time. In most cases, the conditional entropies of all WTA circuits were converged after approximately a couple of rounds of total data presentation (after 448 sec). While the visual observation of spike bursting in the output WTA after learning seemed to show less salient differences than expected, the traces of normalized conditional entropy showed that the final WTA outperformed the individual ensemble WTAs in nearly all cases. Fig 7 shows three particular examples of different gating WTA performances of: (A) better than the ensemble average, (B) similar to the ensemble average, (C) worse than the ensemble average. It is interesting to note that the performance of the gating WTA, which actually guides the whole ensemble, does not have to have the best performance in order for the overall performance of the ensemble to be better than that of the individual classifiers.

As well as supporting the theoretical model of the logical voter ensemble presented earlier, these initial experiments demonstrated that the ensemble architecture for a population of spiking networks successfully extended into a more biologically realistic implementation in which the individual classifiers and the combining mechanism all operated and learned in parallel.

#### SEM ensemble learning with different feature selection schemes

The initial experiments described in the previous section used a simple random input feature selection scheme. In order to investigate the influence of feature selection on learning in the SEM ensemble, a detailed set of experiments were carried out to compare a number of different feature selection heuristics. This section presents the results of those experiments. The basic experimental setup and the visual classification task were the same as in the previous section. In each of the new feature selection schemes, pixel subsets were stochastically selected from controlled probability distributions. Ensemble behaviour was compared across the controlled feature distributions and the random selection scheme in terms of the relationship between performance and ensemble diversity.

**Fig 8:**
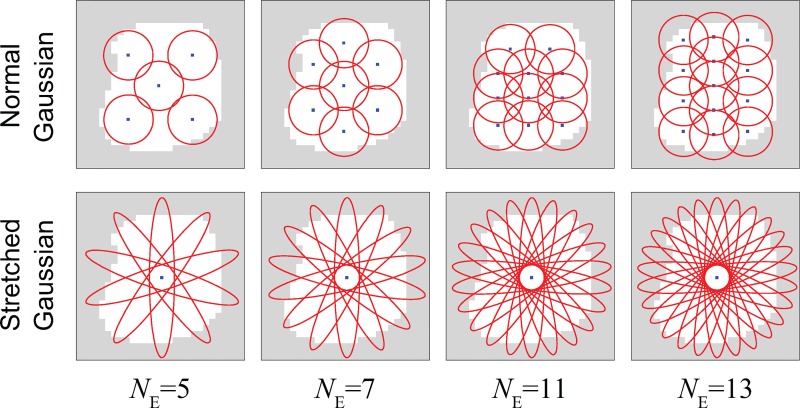
Illustrative images for controlled feature assignment for SEM ensemble networks. White regions indicate available pixels (active region) as defined by preprocessing, and the Gaussian means for the normal Gaussian selection scheme are evenly placed inside such regions by random placement procedure (See Methods for details of the actual Gaussian mean placement). The number of stretched Gaussian features used increases linearly with ensemble size (see Methods for details). The diameters of red circles and ovals roughly represent the full width at a tenth of maximum (FWTM) for each principal direction (the length of an oval is shown far shorter than it actual is for the sake of visualization—long ovals are used to ensure they form roughly uniform bars in the region of available pixels). In all cases, exactly 1/4 of pixels from the available (white) region are stochastically selected (without replacement) for each ensemble network according to each distribution function.

**Fig 9:**
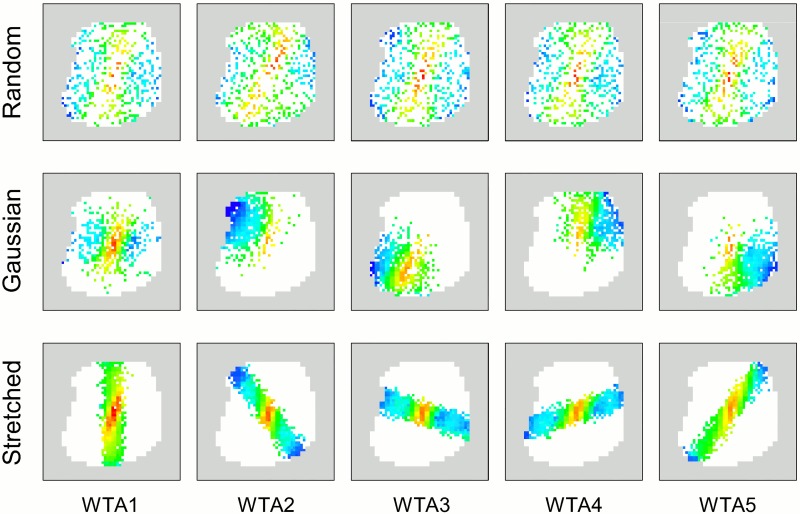
Examples of STDP weight maps from different feature selection schemes when *N*_*E*_ = 5. The weight maps for the ensemble WTA neurons which represent the digit 1 after learning are shown.

For the controlled feature subsets, two Gaussian distribution schemes were tested, being systematically investigated for various ensemble sizes *N*_*E*_. These schemes are reminiscent of basic biological topographic sensory receptive fields/features [45]. In order to promote diversity of input patterns for ensemble members, each distribution was designed to enable pixels to be drawn from different regions of the image, and for each ensemble WTA to receive projections from different input neurons, corresponding to the selected pixel subsets. Hence each of the SEM classifiers in the ensemble received its inputs from a different region of the image as defined by the distributions. The first method selects pixels by sampling from *N*_*E*_ normal 2D Gaussian distributions (i.e. with identity covariance matrices) with different means (mean positions are distributed evenly on the image)—one for each ensemble member. The second Gaussian method uses the same number of stretched Gaussian distributions (the selected pixel group forms a thick bar on the image) all having the same mean at the centre of the image but with varying orientations (which differ by *π*/*N*_*E*_ rad)—see Figs 8 and 9 for illustrative descriptions of each selection scheme and their resultant visual regions. See Methods section for further details of the schemes.

Since the SEM network implements spike-based stochastic EM [30], its solution is only guaranteed to converge to one of the local maxima, dependent on both the initial conditions and the stochastic firing process, which means that the system behaviour can vary to some extent between repeated trials. Thus it was necessary to set some criterion for the comparison of the system behaviours under different feature assignment schemes. The most obvious approach would be to compare them at their peak performances when all ensemble WTAs and the gating WTA are at their global maxima. If all the ensemble and gating WTAs produce their maximum performances, the final result will be also be at the maximum. However, it is hard to manually search all WTAs for maximum performances (which is another optimisation problem), thus we first observed the performance of the system under different conditions only when the performance of the full-featured gating network (i.e. using input from all features as in Fig 6) had reached a level close to its best possible value. Later, more reliable, comparisons were performed by using statistics from a number of repeated trials using supervised output from the gating network by giving the true class labels without learning (thus forcing identical gating network behaviour over trials).

**Fig 10:**
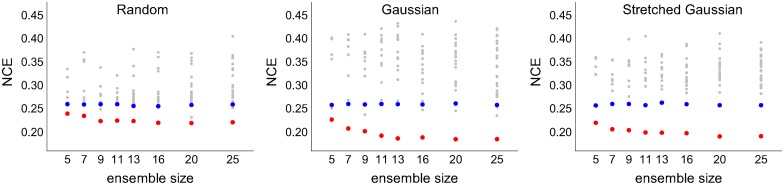
All WTA performances vs. ensemble sizes for different feature selection schemes. Results having similar gating network performances are depicted by manually finding the ‘best’ gating network performances at around NCE≈0.26. All NCE values were taken at the end of simulations which were run for two rounds of input presentations (*t* = 448 sec). Colors represent: ensemble networks (grey), gating network (blue), and the final output network (red).

The ensemble system using three different feature selection scheme (random, normal Gaussian, stretched Gaussian—see Fig 8) was investigated with eight different ensemble sizes *N*_*E*_ = {5, 7, 9, 11, 13, 16, 20, 25}. Fig 10 shows an example of the performances of all WTA outputs after running two rounds of input presentations. In order to minimise the influence of different gating network performances on the comparison of final performances, in the runs summarized in the figure only the results from similar gating network performances were plotted. The gating network always used the same full set of features, and the results from the reasonably high gating network performances (NCE≈0.26) were found by manually repeating several tens of runs with different initial weights. The results show that the ensemble systems using the Gaussian feature selection schemes both outperform, to a similar degree, random selection and that the final performances increase (i.e. NCE decreases) with ensemble size in all cases.

Further trends in system behaviour were investigated in more detail by averaging over repeated simulations using a supervised gating network whose neurons output the true class (the *i*th neuron fires when the image from class *i* is presented), thus taking variability in the unsupervised gating network out of the equation. At the beginning of each image presentation (*t*_0_), a supervised gating neuron output was manually given as a train of three spikes, with an interspike interval of 15ms starting from *t*_0_ + 5ms. By alleviating issues of variability, analysis of repeated simulations with the supervised gating network allowed better insight into the dependency of system behaviour on the feature selection schemes and ensemble size. The mean positions of the Normal Gaussian features were randomly ‘jittered’ about (within constraints, see Methods for details) between simulations so as to eliminate any dependence on exact pre-determined positions. We also measured the diversity of ensemble members in order to investigate its influence on the final performance. Although various measurements for the diversity of classifier ensembles have been proposed, there is no globally accepted theory on the relationship between the ensemble diversity and performance, and only a few studies have conducted a comparative analysis of different diversity measures for classifier ensembles [56, 57]. Among them, we chose an entropy based diversity measure [56, 58, 59] because it is a none-pairwise method (hence less computationally intensive) and has been shown to have strong correlation with ensemble accuracy across various combining methods and different benchmark datasets.

After learning has converged, the diversity of an ensemble of size *N*_*E*_ for *N*_*C*_ classes is calculated over the total input presentations as:

$$\begin{array}{c} \text{Div}=\frac{1}{M}\sum_{l=1}^{M}\sum_{k=1}^{N_{C}}-P_{k}^{l}{\text{log}}_{N_{C}}P_{k}^{l} \end{array}$$ (12)

$$\begin{array}{c} P_{k}^{l}=\frac{1}{N_{E}}\sum_{j=1}^{N_{E}}\frac{n_{k}^{j}\left(d_{l}\right)}{\sum_{i=1}^{N_{C}}n_{i}^{j}\left(d_{l}\right)} \end{array}$$ (13)

where *M* is the number of input data, and $P_{k}^{l}$ represents the proportion of ensemble members which assign *d*_*l*_ to the instructed class *k* given by the gating network. While the original diversity metric simply counts the number of classifiers giving the same decision, the SEM network output consists of multiple spikes which can have originated from different output neurons within the time window of the image presentation. Thus $P_{k}^{l}$ is calculated from a soft decision, where $n_{k}^{j}\left(d_{l}\right)$ is the number of spikes from the neuron of the *j*th ensemble network which represents the *k*th class under the presentation of input data *d*_*l*_. Identifying which ensemble network neuron represents the *k*th class is done by counting the total number of spikes from each neuron when the *k*th gating network neuron fires and assigning the neuron which fires most.

**Fig 11:**
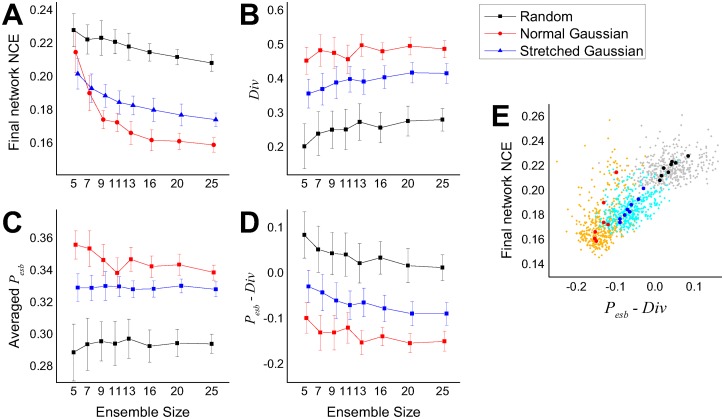
Statistics of ensemble performances and diversities for different feature selection schemes and ensemble sizes. Each point in the graphs (A-D) is the averaged value of 50 simulations, and the error bars represent standard deviations. *E*_esb_ in (C, D, E) represents the average NCE of ensemble members at each simulation, Div (B, D, E) is diversity. (E) Final network NCE vs. the difference of diversity and average ensemble NCE. The background dots (grey, orange, light blue) represent every individual simulation from all three feature selection schemes (random, normal Gaussian, stretched Gaussian respectively) and eight ensemble sizes (3×8×50 = 1200 runs), and the larger dots are the average values of each of 50 repeated simulations (same colors as A-D).

The result from repeated simulations (Fig 11) showed that the normal Gaussian selection scheme provided the best performances even if the average ensemble performance ($E_{\text{esb}}=\frac{1}{N_{E}}\sum_{j}{\text{NCE}}_{j}$) was the worst. As expected, the ensemble diversities showed an inverse relationship with the final network NCE, indicating its crucial role in the combined performance. It can be inferred that while the two Gaussian feature schemes try to select pixel subsets explicitly from different regions of the image, the normal Gaussian scheme generally has more superimposed pixels between subsets than the other. This results in higher redundancy among the output of ensemble members, and hence higher diversity. Preliminary ‘feature jitter’ experiments with higher degrees of noise made it clear that the normal Gaussian scheme works best when the features are reasonably evenly spread over the active region of the image with decent separation between the means—in other words a set of evenly spread reasonably independent features. This fits in with insights on how the architecture works (good performance is encouraged by not too much correlation between individual ensemble member inputs, and a good level of diversity in the ensemble—appropriately used the normal Gaussian features are a straightforward way of achieving this). While performance gets better as the ensemble size increases, diversity roughly increases with ensemble size, indicating a greater chance of disagreement in outputs between ensemble members as the population size increases. Krogh and Vedelsby (1995) [60] have shown that the combination performance (final error *E*) of a regression ensemble is linearly related to the average error ($\bar{E}$) and the ambiguity ($\bar{A}$) of the individual ensemble members as follows: $E=\bar{E}-\bar{A}$, where each term corresponds to the final network performance, average ensemble performance, and diversity in our system. We can expect a similar linear relationships between these quantities and indeed Fig 11E shows the linear relationship between the final network performance and *E*_esb_−Div.

**Fig 12:**
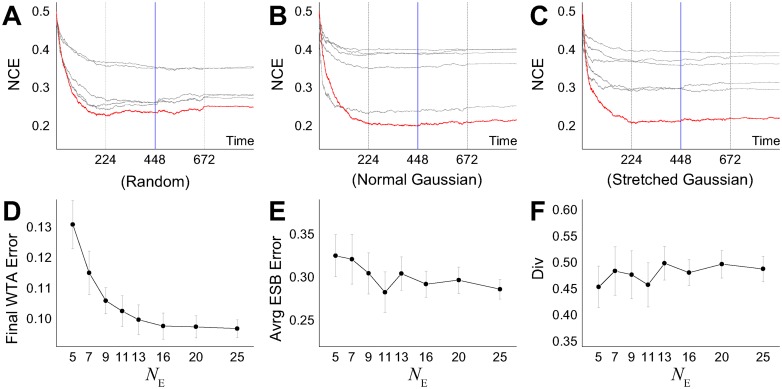
(A, B, C) Training and test performances demonstrating generalization to unseen data (*N*_*E*_ = 5). The testing phase starts at iteration 448 by freezing the weights and by replacing the input samples by the test set which was not shown to the system during the learning phase. (D) Test set error rates of the final output unit, (E) the average ensemble error rates, and (F) the training phase diversities (same as in Fig 11) over different ensemble sizes using the normal Gaussian selection scheme on the integer recognition problem. Each data point was plotted by averaging 50 runs, where the error bar shows the standard deviations. NCE calculations as in Fig 7.

Fig 12A-12C shows that the trained ensemble generalized well to unseen data. Its performance on unseen classification data compared very well with its performance on the training data. In common with all the other earlier results, the figure shows the NCE entropy measure for performance because of the unsupervised nature of the task, where the ‘correct’ associations between input data and most active output neuron are not known in advance. Individual classifier performances are shown in grey, and the overall ensemble (output layer) performance is shown in red. An alternative is to measure the classification error rate in the test phase in relation to the associations between the class of the input data and the output neuron firing rates made during the training phase. In terms of this classification error rate, the trained ensemble typically generalizes to unseen data with an error of 15% or less. The best prediction performances were found using the normal Gaussian selection scheme (Fig 12D–12F), which resulted in an error rate of 10% or less. It can be seen that not only the ensemble size but also its diversity in the training phase influences the performances on the unseen test set, where the generalization performances of ensembles having greater diversities can outperform those with larger ensemble sizes (ex. *N*_*E*_ = 13). Similar trends relating diversities and average test error rates of ensemble members, indicate that networks in the more diverse ensembles are more likely to disagree with each other because of a greater number of misclassifications. However, their combined output eventually yields a better generalization performance on unseen data, indicating that ensemble diversity is more important for the final result than the individual classifier performances.

### Comparison with an STDP Only Ensemble

Although the starting points for the ITDP based ensemble architecture proposed in this paper were the earlier hypotheses about MoE type architectures operating in the brain [6], and the realization that the circuits involved in ITDP studied in [26, 29] had exactly the properties required for an ITDP driven gating mechanism that could control ensemble learning, an alternative hypotheses involves a hierarchical STDP only architecture. A multi-layered STDP system where the final layer learns to coordinate the decisions of an earlier layer of classifiers might also provide a mechanisms for effective ensemble learning.

The SEM neural network classifiers realize expectation maximization by learning the co-firing statistics of pre and postsynaptic neurons via STDP. The neurons of the input layer represents discrete-valued multidimensional data (ex. digital pixel image) using a spike-coded vector, where the value of each dimension is expressed by a group of exclusively firing neurons representing its corresponding states. Since the spike output of a WTA ensemble similarly can be regarded as the binary-coded multidimensional input data for the final layer (ex. *N*_*E*_ dimensional data where the value of each dimension has *N*_*C*_ states), this naturally leads to the possibility that the latent variable (hypothesis) of a given ensemble state can be inferred by the final WTA network using STDP learning instead of ITDP. One difference between the ensemble WTA layer and the input layer during the presentation of input data is that the firing probabilities of WTA neurons are not exclusive for a given input sample (more than one neuron can have a non-zero firing probability), while the population code used in the input layer neurons always have all-or-none firing rates, which means that the state of the given input data is represented stochastically in the WTA layer. Although, as a form of interference, this might inherently affect the behavior of a SEM network, previous work [30] indicates that it should still be able to deal with incomplete or missing data.

Possible applications of multi-layered SEM microcircuit were suggested in [30], and a further study [61] has shown the power of recurrent networks of multiple SEM circuits when used as a neural reservoir for performing classification, memorization, and recall of spatiotemporal patterns. These insights suggest an STDP only implementation of the MoE type architecture presented earlier might be viable. Hence we conducted a preliminary investigation of using STDP to learn the second layer connection weights (i.e. connections between the ensemble and final layer, Fig 4), making a comparison of the use of STDP and ITDP in that part of the ensemble classifier system.

The learning of the second layer of weights by STDP was done straightforwardly by applying the same learning rule as in the first layer connections (between the input and ensemble layers). All other settings and parameters were exactly the same as the original system. In order to avoid the influence of the inevitable trial-to-trial variance of the presynaptic SEM ensemble when the two learning rules are tested separately, the original ensemble network architecture was expanded by having two final (parallel) WTA circuits which both receive connections from the same ensemble WTAs, but are subject to different synaptic learning rules (one for STDP and the other for ITDP). This setup, where the learning rules are tested in parallel, ensures that both final layer WTAs receive exactly the same inputs, so that any differences in their final performances depend only on the different synaptic learning rules. For the repeated simulations with the normal Gaussian feature selection scheme, the same initial mean positions were used without the random mean placement ‘jittering’. This is because the purpose of the current experiment is to compare the two plasticity methods under as identical conditions as possible, and we know from the earlier experiments with the ITDP ensemble that the performance and trends of the fixed normal Gaussians was very close to the average of the randomly jittered placements. These procedures enables a well-defined, unbiased comparison between the two learning rules.

The connections from the gating WTA to the ITDP final layer operate exactly as in the experiments described earlier (i.e. as genuine gating connections involved in the heterosynaptic plasticity process). For comparability, the STDP final layer also receives projections from the gating WTA, but they of course operate very differently—they are just like the connections to the final network from any of the ensemble networks. Therefore in the STDP case the gating WTA does not have an actual gating function but effectively operates as an extra ensemble member. The corresponding synaptic weights are learnt by STDP in just the same way as for all other ensemble WTA projections to the STDP final layer neurons. This use of an additional ensemble member is potentially advantageous for the STDP final network in terms of the amount of information used.

**Fig 13:**
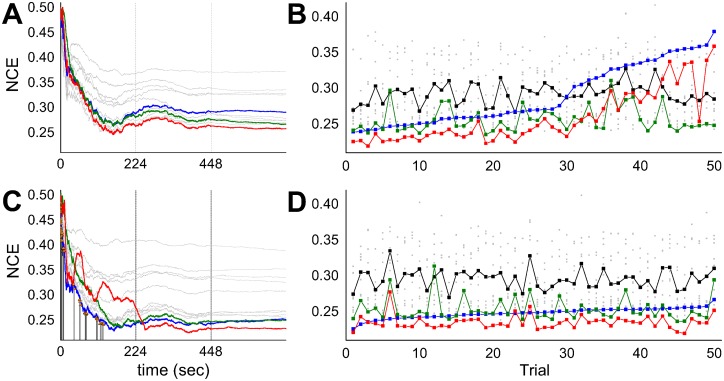
Training performances of the expanded STDP/ITDP networks (using random feature selection on the MNIST handwritten digits classification task as in earlier experiments). Each color represents, red: ITDP final WTA, green: STDP final WTA, blue: gating WTA, grey/black: ensemble WTAs and their average. (A, B) An example of time courses of performances and the final performances from 50 repeated trials using unsupervised gating WTA. The individual trials were sorted by gating WTA performances in ascending order. (C, D) Simulations using the automatic selection of gating WTA. The vertical lines with arrowheads in C indicate where the switching of gating WTA occurs (see text for further details).

The results of multiple runs of the expanded comparative architecture on the MNIST handwritten digits recognition task with random feature selection are illustrated in Fig 13. It is clear from these initial tests that the STDP version compares favourably with the ITDP version, although is generally not as good. The performance of the STDP final WTA over repeated trials shows that on many runs it outperforms most of the ensemble WTAs (i.e. ensemble learning is successful in this version of the architecture). Although the STDP net is capable of bringing improved classification from the SEM ensemble, its performance variance over repeated trials is higher than the ITDP net, indicating less robustness against the various ensemble conditions. However, while the ITDP net is dependent on the gating WTA performance (as we know from earlier experiments—Fig 7), no single presynaptic WTA circuit strongly influences the STDP net performance. The result of repeated runs sorted by the gating WTA performance (Fig 13B) indeed shows this dependency of the ITDP net, and the STDP net outperforms the ITDP net in the region where the gating WTA performances are the worst. However, as was shown with earlier experiments, it is relatively easy to find good initial gating network settings, and it might not be unreasonable to assume these would be partially or fully hardwired in by evolution in an ITDP ensemble. The dependence of ITDP on (a reasonable) gating signal may be disadvantageous in terms of the performance consistency in this type of neural system in isolation, and without any biases in initial settings, but on the other hand, the gating mechanism (which after all is the very essence of the ITDP system) can act as an effective and compact interface for providing higher control when connected to other neural modules. For example, the supervising signal could be directly provided via a gating network from the higher nervous system, or the gating signal could be continuously updated by reward-based learning performed by an external neural system such as the basal ganglia. Also it is possible that multiple ITDP ensemble modules could be connected such that the final output of one module is fed to the gating signal of other modules (similar to the multilayered STDP SEM networks), achieving successive improvements of system performance as information is passed through modules.

Fig 13C and 13D show the performances using a high performing ensemble WTA as the gating WTA which is automatically selected during the early simulation period. The gating WTA was continuously updated during the first round of dataset presentation (0 < *t* < 224) by assigning one of the ensemble WTAs as the gating WTA whenever the current gating WTA is outranked by it. This procedure was used, rather than assigning previously found good (ITDP) gating network settings, in an attempt not to potentially bias proceeding against STDP by using a network known to be good for ITDP. When the gating WTA is replaced by the selected ensemble WTA, the indices of its neurons representing corresponding classes also changes. Thus the entire set of ITDP weights are automatically re-learnt to new values, which causes the transition in the NCE value of the final WTA until re-stabilization (the hills in the red line in Fig 13C).

**Fig 14:**
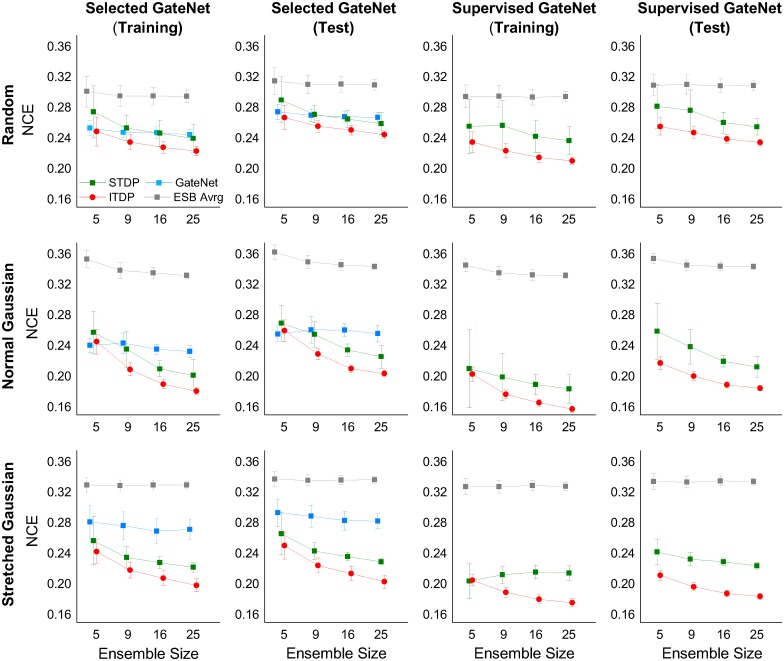
Average performances of STDP and ITDP ensembles over 50 trials on the MNIST handwritten digits task using selected/supervised gating WTAs for different feature selection schemes and ensemble sizes (*N*_*E*_ = 5, 9, 16, 25). The training and test phases were run for three and two rounds of dataset presentation respectively. The error bars represent the standard deviations of the performances from corresponding repeated runs.

Indeed, we can see from the results of the more detailed set of comparative experiments shown in Fig 14 that given a qualified gating signal of the kind describe above (i.e. from a gating network that performs classification reasonably well), the ITDP final net consistently and significantly outperformed the STDP final net over a wide range of conditions (feature selection scheme, ensemble size) in both training and testing. This was the case even though the STDP net uses one more presynaptic WTA circuit ensemble member, which can be seen to confer an advantage (first two columns in Fig 14). Clearly, if the gating network was used only in the ITDP case, and the main ensemble was the same size under both conditions, then the ITDP version’s margin of superiority would be increased further.

It is interesting to note that the overall trends of the final performances of both methods are similar to each other over the repeated trials in the region of good performance gating WTAs (the ups and downs of the red and green plots over the trials in Fig 13B and 13D follow each other quite closely). There is also a similar dependency of the average performances on the ensemble sizes (Fig 14), which suggests that there might be some shared underlying mechanisms in both combining methods. In the STDP ensemble, the synapses carrying the presynaptic spikes onto the postsynaptic neurons get enhanced after a few milliseconds of neural firing. Since all the WTA neurons fire highly synchronous bursts of spikes during every input presentation (the behavior is similar to the clocked output of the abstract voter ensemble model), in most cases the last spike of the final WTA burst triggering STDP follows right after the end of the presynaptic bursts. This leads to the synaptic potentiation by STDP reflecting all the presynaptic bursts. Considering the plasticity curves of STDP and ITDP in our model are of a similar type with a few tens of milliseconds of time shift, both plasticities can be generally thought as enhancing the synaptic weight if two neuron co-fire around the peak of the curve, and depressing it otherwise. This insight leads to the hypothesis that the final WTA in the STDP network acts functionally quite similarly to the gating WTA in the ITDP network. Among the presynaptic ensemble WTA neurons, the better performing neurons (those which fire only under the presentation of a specific class) will fire more spikes than the worse performing neurons. This is because the neurons of each ensemble WTA typically fire highly regular burst of 3-4 spikes in total. The best performing neurons in the ensemble layer fires all 4 spikes for its corresponding class and remains silent for the other classes. In the poorer performing WTA neurons, more than two neurons will fire 1-2 spikes each, resulting in the dispersion of spikes. Thus, over the course of STDP weight updates, the weights from the better performing presynaptic WTA neurons will get more potentiation (by summing EPSPs from all 4 presynaptic spikes) than the connection weight from the more poorly performing neurons (which typically carry only 1-2 spikes). This leads us to infer that the best performing presynaptic WTA neurons under each class presentation generally influence the final WTA most as learning proceeds (through the Hebbian STDP process). This autonomously drives the final WTA towards better performance through increased correlated activity with the ensemble, effectively making it a good ‘gating’ WTA (or at least ‘guiding’ WTA). This ‘guiding’ results in better performance of the combined ensemble output in an analogous way to the explicit gating signals in the ITDP ensemble mechanism. Of course the STDP version requires correlated pre- and post-synaptic firing from the start in order to gain traction, whereas the more direct ITDP version does not require post-synaptic firing. Although this STDP ‘gating signal’ may result in positive feedback of the final WTA behavior, inputs from the other presynaptic neurons always interfere with it, preventing an indefinite increase of system performance. The effect of supervised gating signals shown in Fig 14 indeed shows the difference between the two mechanisms: the STDP final net has increased performances driven by the supervised signal from one of the presynaptic WTAs during the training phase, but its performance drop is much larger than for the ITDP final net in the test phase after the supervised signal is removed. In particular, the odd dependence of the STDP net on ensemble size in the stretched Gaussian selection case (where performance decreases with ensemble size in the training phase, instead of increasing as in all other cases, and the discrepancy with the test phase is particularly marked: Fig 14 bottom of 3rd column) indicates the possibility of a negative effect of the supervised signal when the ensemble size is small, where the training result can be deceptive because of the large influence of the supervising signal on the final WTA relative to the inputs from the rest of the presynaptic WTAs. By contrast the explicit gating signal in the ITDP system is more stable and less prone to such effects, providing better overall performance.

## Discussion

**Fig 15:**
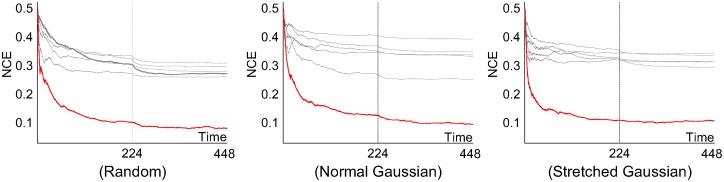
Training performances of ensemble networks using different datasets for each ensemble member (*N*_*E*_ = 5). Individual classifier performances are shown in grey, and the overall ensemble (output layer) performance is shown in red. Results are for various input feature selection schemes on the handwritten integers problem as in the previous section.

The main aim of this paper was to explore a hypothesized role for ITDP in the coordination of ensemble learning, and in so doing present a biologically plausible architecture, with attendant mechanisms, capable of producing unsupervised ensemble learning in a population of spiking neural networks. We believe this has been achieved through the development of an MoE type architecture built around SEM networks whose outputs are combined via an ITDP based mechanism. While the architecture was successfully demonstrated on a visual classification task, and performed well, our central concern in this paper was not to try and absolutely maximize its performance (although of course we have striven for good performance). There are various methods and tricks from the machine learning literature on ensemble learning that could be employed in order to increase performance a little, but a detailed investigation of such extensions is outside the scope of the current paper, making it far too long, and some would involve data manipulation that would move the system away from the realms of biological plausibility, which would detract from our main aims. However, one interesting direction for future work related to this involves using different input data subsets for each ensemble member. This can increase diversity in the ensemble which has been shown to boost performance in many circumstances [18, 49], a finding that seems to carry over to our spiking ensemble system according to the observations on diversity described in the previous section. Preliminary experiments were carried out in which each SEM classifier was fed its own distinct and separate dataset (all classifiers were fed in parallel, with an expanded, separate set of input neurons for each classifier, rather than them all using the same ones as in the setup described earlier in this paper). A significant increase in the overall ensemble performance after training was observed as shown in Fig 15. Further work needs to be done to investigate the generalization of these results and to analyse differences in learning dynamics for the ensemble system with single (one set for all classifier) and multiple (different sets for each classifier) input data sets. The issue of how such multiple input data sets might impinge on biological plausibility must also be examined. A related area of further study is in applying the architecture to multiple sensory modes, with data from different sensory modes feeding into separate ensemble networks. Some of the biological evidence for ensemble learning, as discussed in the Introduction section, appears to involve the combination of multiple modes. Although we have tested the architecture using a single sensory mode, there is no reason why it cannot be extended to handle multiple modes.

While our SEM ensemble model mimics the general MoE architecture, the overall process is not identical to that used in the classic MoE system [18, 24]. A key difference is that the operation of the SEM gating WTA on the ensemble outputs is not based on immediate training input but is accumulated by slow additive synaptic plasticity over a relatively long time scale, whereas the standard MoE gating network instantaneously changes the strength of ensemble efferents for each input vector. Therefore our spiking system is not as adept at directly and quickly filtering out the wrong output from the ensemble WTAs when an output neuron in the ensemble fires for multiple classes. In this case the false spikes are also passed to the final layer through the enhanced connections. However, because such a neuron has a higher probability of firing for multiple classes, it dissipates its synaptic resource over multiple efferent connections, resulting in lower synaptic weights than in the case of a neuron which fires predominantly for one class. Hence the neuron that fires for multiple classes has less chance of winning at the final output layer WTA. Similarly, false spikes from the gating WTA will result in less chance of enhancing the corresponding target set of ITDP weights because of timing mismatch. In this way our spiking ensemble system can effectively filter out these false classifications, but using different learning dynamics from the classical system. However, if a large number of ensemble WTAs fire equally wrongly for the same class, the final output develops a higher chance of generating the wrong output. The standard architecture can of course suffer from the same problem [18, 49]. This can happen, for instance, when two input images are hard to discriminate (such as the digits 3 and 8), even if the input subfeatures are randomly selected. Therefore the system is not entirely free from the feature selection problem as experienced in other ensemble learning methods. This limitation meant that in such circumstances simulations using high ensemble sizes did not significantly improve the overall performance (Fig 11), indicating a lack of ensemble diversity. Preliminary experiments indicated that by using an evolutionary search algorithm to evolve individual feature selection schemes for each ensemble member, diversity is increased, alleviating this problem greatly and significantly increasing performance. This is reminiscent of individually evolved receptive fields/input ‘features’ for spatially separated networks in the cortex and other areas. Future work will explore this issue more thoroughly. An interesting extension is the possibility of a form of evolutionary search being neuronally integrated into the current architecture [62] so that feature selection is performed in parallel with the other plastic processes, becoming part of the overall adaptation.

The empirical work on which we base our ITDP model [26, 29] was conducted *in vitro*. While this was of course because of the technical difficulty of conducting such research *in vivo*, it should be noted that work by Dong et al. (2008) [35] suggests that in some circumstances there can be activity dependent differences in the dynamics of heterosynaptic plasticity operating *in vivo*. While Dong et al. were looking at heterosynaptic plasticity in the hippocampus, they were not studying ITDP as defined in [26] and they were observing quite different neural pathways from Dudman et al. (specifically, Dong’s system involved Schaffer and commissural pathways, crucially without the different proximal and distal projections onto CA1 found in Dudman’s system, from EC and CA3 respectively—instead the two CA1 inputs are both from CA3). However, Dong et al. (2008) [35] made the interesting finding that in the system they were studying, *in vivo*, coincident activity of converging afferent pathways tended to switch the pathways to be LTP only or LTP/LTD *depending* on the activity states of the hippocampus [35]. If such findings extended to the system we have based our learning rule on, then of course our hypothesis would have to be revised. We are working under the assumption that the behaviour is stable *in vivo*. Recently Basu et al. (2016) [29] have provided some indirect evidence that the ITDP behaviour of the particular circuits we are basing our functional model on does hold *in vivo*. They cite studies of the temporal relation of oscillatory activity in the entorhinal cortex and the hippocampus *in vivo* that suggest that the disinhibitory gating mechanism enabled by the LRIPs may indeed be engaged during spatial behavior [63, 64] and associational learning [65]. For example, during running and memory tasks, fast gamma oscillations (100Hz) arising from EC are observed in CA1 and precede the slow gamma oscillations (50Hz) in CA1, which are thought to reflect the CA3 pyramidal neuron input [63]. Crucially, EC-CA1 gamma activity and CA3-CA1 gamma activity display a 90° phase offset during theta frequency oscillations (8 to 9Hz) [63] which is consistent with a 20-25ms time delay. However, since any ensemble learning of the kind we have presented here would be part of a wider ‘cognitive architecture’, it is interesting to speculate that some activity dependent influence on the dynamics of such learning might occur in the bigger picture (e.g. moderating or switching on/off ensemble learning in certain conditions).

For reasons discussed earlier in this paper, ITDP seems a very good candidate for involvement in a biological mechanism ideal for combining ensemble member outputs, but it was naturally interesting to also attempt an all STDP implementation. Although we had imagined interference effects would compromise its learning ability, this version of the architecture performed surprisingly well. When the gating network performed relatively poorly, the STDP version compared very favourably with the ITDP version. However, with good (or at least reasonably) performing gating networks the ITDP version was significantly better over all conditions. This highlighted the dependence of the ITDP architecture on a gating network that achieves reasonable performance in agreement with the similar findings from the initial more abstract (voter) model. This shows that there is a small price to pay for the advantage the ITDP process confers, namely that it strengthens connections without a need for the corresponding final output neuron to be firing, thus providing a strong guiding function. The various methods for reducing this reliance (or at least ensuring the gating performance is always reasonable) that were outlined in the previous section will be the subject of future work. Preliminary analysis, as discussed in the previous section, suggests that there are some very interesting parallels between the ways the successful ITDP and STDP architectures operated, notably that the best performing ensemble WTA neurons in the STDP version had a guiding role functionally similar to that of the gating network in the ITDP version. While the differences and commonalities between ITDP and STDP dynamics in combining ensemble classifiers were briefly discussed in relation to the preliminary experiments, a more thorough comparative analysis of the effects of various conditions on both learning schemes will be addressed in the future work. Certainly the ITDP vs STDP work undertaken so far suggests that STDP-only architectures are another plausible hypothesis for ensemble learning in populations of biological spiking networks.

Lateral inhibition in the SEM networks—which provides the competition mechanism in the WTA circuits—is modeled as a common global signal that depends on the activity of the neurons in the network [30]. This effectively models a form of strong uniform local lateral inhibition as widely experimentally observed in the cortex [66, 67]. This inhibition mechanism is a core part of the SEM network dynamics and reflects the fact that they are small locally organised networks. We assume multiple such networks act as the ensemble members in our architecture. However, it might be possible to model the ensemble layer by a bigger single group of neurons which inhibit each other according to a ‘Mexican hat’ type function. Since with this form of inhibition (which is also commonly assumed [68]) the effect drops off with distance, with strong interaction among nearby neurons, a set of overlapping networks could emerge that function similarly to a smoothed version of multiple WTA circuits.

Dealing with arbitrary (unknown) numbers of classes with our ITDP ensemble architecture in a general unsupervised manner is a challenging future direction, although an individual SEM network with a sufficient number of output neurons has been shown to perform unsupervised clustering of a given dataset to some extent [30]. It might be possible to employ a higher control to vary the number of classes in a supervised way as shown in [72]. More preferably, the smoothed version of a lateral inhibition mechanism using the Mexican hat topology may be capable of dealing with unknown numbers of classes in a more biologically plausible way by incorporating more sophisticated synaptic and neuromodulatory mechanisms.

The novel architecture presented here demonstrates for the first time how unsupervised (or indeed any form of) ensemble learning can be neurally implemented with populations of spiking networks. Our results show that the ensemble performs better than individual networks. The lack of diversity within the population, which sometimes becomes apparent, will be tackled in the next phase of work as outlined above. It is also possible that the relative strength of the ensemble method, in terms of efficiency of learning, might change when reducing the time spent on learning in the SEM networks (i.e. there may be an interesting resource/performance trade-off). This issue will also be investigated.

## Methods

### Analytic Ensemble Model

#### Derivation of expression for synaptic weights under the influence of ITDP

From Eqs 1–4, we derived the expected value of the weight *w* at equilibrium under constant presynaptic firing probabilities to give the expression in Eq 5 as follows.

$$\begin{array}{c} \text{E}\left[\Delta w\right]=\left(ae^{-w}-1\right)p\left(m\right)p\left(g\right)+\left(-1\right)\left(p\left(m\right)+p\left(g\right)-2p\left(m\right)p\left(g\right)\right)+\left(0\right)\left(p\left(\neg m\wedge \neg g\right)\right)=0 \end{array}$$ (14)

Solving for *w* progresses thus:

$$\begin{array}{c} e^{-w}=\frac{1}{a}\left(\frac{p(m)+p(g)-p(m)p(g)}{p(m)p(g)}\right) \end{array}$$ (15)

Taking logs on both sides of Eq 15 to pull out *w* gives

$$\begin{array}{c} {w|}_{\text{E}[\Delta w]=0}=\text{log}\left(a\right)+\text{log}\left(\frac{p(m)p(g)}{p(m)+p(g)-p(m)p(g)}\right) \end{array}$$ (16)

This gives the expected value of *w* at equilibrium expressed in terms of the two probabilities *p*(*m*) and *p*(*g*).

#### Analytic solution of ITDP weights

In practice, a voter in our analytic, abstract ensemble model emulates an abstract classifier which is assumed to have been fully trained in advance using an arbitrary set of input data. A typical expression of the statistical decision follows the Bayesian formalism, where the firing probability of each voter neuron *m*_*i*_ represents the posterior probability *p*(*m*_*i*_|**x**) of the corresponding class label for a given input vector. The input vectors for each ensemble voter are distinct measurements of the raw input data (e.g. determined by using different feature subsets for each voter). A voter outputs a decision with probability one (∑_*i*_ *p*(*m*_*i*_|**x**) = 1) by exclusively firing one of its neurons according to sWTA mechanism. We assume that the input measurements for different voters ensure the ideal diversity of the ensemble so that the decision outputs of voters are independent of each other. We set the number of neurons in a voter to the number of possible decisions (classes) *N*_*C*_; the firing probabilities of the neurons for the presented sample comprises a probability vector. The probability vectors of a voter are defined differently for each sample, comprising *M* probability vectors of size *N*_*C*_ where *M* is the number of data samples and *N*_*C*_ is the number of existing classes (equal to the number of voter output neurons). The statistics of probability vectors for each pattern class are designed differently in order to emulate the classification capability of voters which is assumed to be fully learnt in advance.

The analytic solution for ITDP learning for the ensemble system is similar to the previous three node formulation, as each connection weight of the ensemble network is estimated by assuming zero expected value of weight change once equilibrium has been reached. Recall the weight update Eqs 4 and 14, which are now written as the sum of the weight changes made from each presented input sample. Consider the probability of sample presentations for *x*_*l*_ during ITDP learning as *p*(*x*_*l*_), where $\sum_{l=1}^{M}p\left(x_{l}\right)=1$. The expected change of individual weights by ITDP can be written as the sum of all long term weight changes occurring at each sample presentation in the same way as in Eq 14,

$$\begin{array}{c} \text{E}\left[\Delta w_{ki}^{j}\right]=\left(ae^{-w_{ki}^{j}}-1\right)\sum_{l=1}^{M}p\left(x_{l}\right)p\left(m_{i}^{j}|x_{l}\right)p\left(g_{k}|x_{l}\right)-\sum_{l=1}^{M}p\left(x_{l}\right)\{p\left(m_{i}^{j}|x_{l}\right)+p\left(g_{k}|x_{l}\right)\\ -2p\left(m_{i}^{j}|x_{l}\right)p\left(g_{k}|x_{l}\right)\}=0 \end{array}$$ (17)

where $p(m_{i}^{j}|x_{l})$ is the firing probability of the *i*th neuron of the *j*th ensemble voter for input sample *x*_*l*_, $w_{ki}^{j}$ is the weight from $m_{i}^{j}$ to the *k*th neuron (*f*_*k*_) of the final voter, and *p*(*g*_*k*_|*x*_*l*_) is the firing probability of the corresponding gating voter neuron which projects to *f*_*k*_. Assuming the constant probability of every sample presentation and solving for $w_{ki}^{j}$ at its equilibrium gives the following analytic solution of weight convergence:

$$\begin{array}{c} w_{ki}^{j}=\text{log}\left(a\right)-\text{log}\left(\frac{\sum_{l=1}^{M}\left\{p\left(m_{i}^{j}|x_{l}\right)+p\left(g_{k}|x_{l}\right)\right\}}{\sum_{l=1}^{M}p\left(m_{i}^{j}|x_{l}\right)p\left(g_{k}|x_{l}\right)}-1\right) \end{array}$$ (18)

where the constant probability of sample presentation *p*(*x*_*l*_) = 1/*M* has been eliminated from the equation.

#### Analytic solutions of final voter firing probabilities

While it is sufficient to express the behaviours of the ensemble voters and the gating voter using pre-determined firing probabilities for the purpose of obtaining weight convergence, the firing probabilities of neurons in the final voter are calculated by integrating the ‘EPSP’s from all presynaptic spikes. Taking the stochastic winner-takes-all Poissonian spiking formulation [30], the firing probability of neuron *k* of the final voter at a discrete time *t* is written as:

$$\begin{array}{c} p(f_{k}\left(t\right))=\frac{e^{u_{k}\left(t\right)}}{\sum_{i=1}^{N_{C}}e^{u_{i}\left(t\right)}} \end{array}$$ (19)

$$\begin{array}{c} u_{k}\left(t\right)=\sum_{j=1}^{N_{E}}\sum_{i=1}^{N_{C}}w_{ki}^{j}\sum_{s}\epsilon \left(t-t_{i,s}^{j}\right) \end{array}$$ (20)

where *u*_*k*_(*t*) is the EPSP of the final voter neuron *k* at time *t*, *N*_*E*_ is the ensemble size, and $t_{i,s}^{j}$ is the time of the *s*’th spike output by neuron $m_{i}^{j}$. The EPSP response kernel *ϵ*(*t*) could be simply modelled as a rectangular function which integrates all the past spikes within a finite time window, or we could use exponential decay to smoothly decrease the potential. However, for the sake of computational convenience for understanding the analytic solution of long term final voter behaviour, we only integrate the instantaneous presynaptic spikes, which is equivalent to using a unit impulse function for *ϵ*(*t*), where all the spiking events are clocked at every discrete time instance as assumed in the voter ensemble system. The average values of the final voter probabilities can be calculated by solving the expected values of time-varying final voter probabilities themselves. At each discrete time *t*, the state of the ensemble is always defined by the firing of *N*_*E*_ neurons from the ensemble voters (one of the *N*_*C*_ neurons fires in each voter), resulting in $N_{S}=N_{C}^{N_{E}}$ possible states of the ensemble. Given a set of ensemble firing states *S* = {*s*_1_, *s*_2_, …, *s*_*N*_*S*__}, let us define an index function *R*(*q*, *j*) which gives the index of the firing neuron of the voter *j* at the ensemble state *s*_*q*_. The function can be defined to return *d* + 1 where *d* is the *j*’th digit of the *N*_*C*_-ary number which is equivalent to decimal number *q*. For example, if *N*_*C*_ = 4 and *N*_*E*_ = 3, then *R*(25, 2) = 2 + 1 = 3 (25 is 121 as a quaternary number). Using this index function, the probability of the occurrence of the state *s*_*q*_ under the presentation of sample *x*_*l*_ can be written very succinctly as a joint probability of neurons firing:

$$\begin{array}{c} p\left(s_{q}|x_{l}\right)=\prod_{j=1}^{N_{E}}p\left(m_{R(q,j)}^{j}|x_{l}\right) \end{array}$$ (21)

The weighted sum of spikes from the ensemble in state *s*_*q*_ arriving at the postsynaptic neuron *k* is

$$\begin{array}{c} u_{k}\left(q\right)=\sum_{j=1}^{N_{E}}w_{kR(q,j)}^{j} \end{array}$$ (22)

The probability of a final voter neuron *p*(*f*_*k*_|*x*_*l*_) at ensemble state *q* is then calculated as in Eq 19. Now we can calculate the expected probability of a final voter neuron under the presentation of sample *x*_*l*_ as:

$$\begin{array}{c} \text{E}\left[p\left(f_{k}|x_{l}\right)\right]=\sum_{q=1}^{N_{S}}\left(p\left(s_{q}|x_{l}\right)\cdot \frac{e^{u_{k}\left(q\right)}}{\sum_{l=1}^{N_{C}}e^{u_{l}\left(q\right)}}\right). \end{array}$$ (23)

The expected firing probability of the final net neuron *k* under the presentation of the samples from class *c* can be written as follows by the law of total probability:

$$\begin{array}{c} p\left(f_{k}|c\right)=\frac{1}{M_{c}}\sum_{l=1}^{M_{c}}p\left(f_{k}|x_{l}\right) \end{array}$$ (24)

$$\begin{array}{c} \text{E}\left[p\left(f_{k}|c\right)\right]=\frac{1}{M_{c}}\sum_{l=1}^{M_{c}}\text{E}\left[p\left(f_{k}|x_{l}\right)\right] \end{array}$$ (25)

This gives the Eq 7, the expected (long term) firing probability of final net neuron *k* under the presentation of class *c*.

#### Simulation of voter ensemble network

The detailed methods for the iterated simulation of the simple analytic spiking ensemble system are as follows.

During the learning phase, the input classes for the ensemble and gating voters were equally presented by turn, which led to the same presentation probability of every input class *p*(*c*) = 1/*N*_*C*_. Consider the input dataset as being divided into *N*_*C*_ subsets belonging to each class; *X*_*c*_ = {*x*_1_, *x*_2_, …, *x*_*n*_, …, *x*_*M*_*c*__} where *c* = 1, 2, …, *N*_*C*_. The following steps were performed at each timestep *t* = (1, 2, …, *T*) with the learning rate *η* = 0.001 and the shift constant *a* = *e*^5^.

- Present a sample *x*_*n*_ from the subset *X*_*c*_, where *n* = {(*t* − 1) div *N*_*C*_} + 1 and *c* = {(*t* − 1)mod*N*_*C*_} + 1.
- All ensemble voters and gating voter fire according to their firing probabilities $p(m_{i}^{j}|x_{n})$ and *p*(*g*_*k*_|*x*_*n*_).
- All weights are updated by ITDP as *w* ← *w* + *ηΔw*. For every weight $w_{ki}^{j}$, $\Delta w_{ki}^{j}=ae^{w_{ki}^{j}}-1$, if both the ensemble neuron $m_{i}^{j}$ and the gating neuron *g*_*k*_ fire. If only one of those two fires, decrease the weight by -1. If neither of them fires, do nothing.

The measuring phase was run for every *X*_*c*_, each for the duration of *T*_*m*_ = *T*/*N*_*C*_, in order to see how well one of the neurons in the final voter fired exclusively for each class. The measuring phase for each class presentation proceeded as follows:

- All ensemble voters and the gating voter fire according to their firing probabilities $p(m_{i}^{j}|x_{n})$ and *p*(*g*_*k*_|*x*_*n*_).
- Each final voter neuron fires after calculating its firing probabilities according to the weighted integration of all presynaptic spikes as in Eq 19.

In order to compare the final voter output from the measuring phase with the analytic solution, we calculate all the momentary probabilities of each final voter neuron during simulation and check their averages with Eq 7.

#### Performance measure

The NCE of a voter is calculated over the input set as:

$$\begin{array}{c} \text{NCE}=\frac{H(C|F)}{H(C,F)} \end{array}$$ (26)

where *C* = {*c*_1_, *c*_2_, …, *c*_*N*_*C*__} is the class of presented inputs, and *F* = {*f*_1_, *f*_2_, …, *f*_*N*_*C*__} denotes the discrete random variable defined by the firing probabilities of the voter neurons *f*_*i*_ for each input class, and *H* is the standard entropy function. NCE can be expressed in terms of the joint probability distribution *P*(*C*, *F*), which has *N*_*C*_×*N*_*C*_ elements, as follows:

$$\begin{array}{c} H(C|F)=-\sum_{n=1}^{N_{C}}\sum_{i=1}^{N_{C}}p\left(c_{n},f_{i}\right)\text{log}\left(\frac{p(c_{n},f_{i})}{\sum_{l=1}^{N_{C}}p\left(c_{l},f_{i}\right)}\right) \end{array}$$ (27)

$$\begin{array}{c} H(C,F)=-\sum_{n=1}^{N_{C}}\sum_{i=1}^{N_{C}}p\left(c_{n},f_{i}\right)\text{log}\left(p(c_{n},f_{i})\right) \end{array}$$ (28)

where we can analytically calculate *p*(*c*_*n*_, *f*_*i*_) from a probability table defined as in Fig 3 or it can be measured from a numerical simulation by counting all the spikes over the simulation.

### SEM Network Ensemble Learning

The detailed methods for the full SEM-ITDP ensemble architecture are as follows.

#### Bayesian dynamics

According to the formulation given in [30], the overall network dynamics can be explained in terms of spike-based Bayesian computation. The combined firing activity of all *z* neurons in a WTA circuit can be expressed as the sum of *K* independent Poisson processes, which represents an inhomogeneous Poisson process of the WTA circuit with rate:

$$\begin{array}{c} R\left(t\right)=\sum_{k=1}^{K}r_{k}\left(t\right). \end{array}$$ (29)

In an infinitesimal time interval [*t*, *t* + *dt*], the firing probabilities of a WTA circuit and its neurons are *R*(*t*)*dt* and *r*_*k*_(*t*)*dt* respectively. Thus if we observe a neural spike in a WTA circuit within this time interval, the conditional probability that this spike originated from neuron *z*_*k*_ is expressed as

$$\begin{array}{c} q_{k}\left(t\right)=\frac{r_{k}\left(t\right)dt}{R(t)dt}=\frac{e^{u_{k}\left(t\right)}}{\sum_{j=1}^{K}e^{u_{j}\left(t\right)}}. \end{array}$$ (30)

Thus a firing event of *z*_*k*_ can be thought as a sample drawn from the conditional probability *q*_*k*_(*t*) which is equivalent to the posterior distribution of hidden cause *k*, given the evidence represented by the input neuron activation vector **y**(*t*) = {*y*_1_(*t*), *y*_2_(*t*), …, *y*_*n*_(*t*)} under the network weights **w**. By following Bayes’ rule, we can identify the network dynamics as a posterior probability which is expressed using prior and likelihood distributions as:

$$\begin{array}{c} p\left(k|y\left(t\right),w\right)=q_{k}\left(t\right)=\frac{e^{w_{k0}}\cdot e^{\sum w_{ki}y_{i}}}{\sum_{j=1}^{K}e^{w_{j0}+\sum w_{ji}y_{i}}}\Rightarrow \frac{p(k|w)p(y|k,w)}{p(y|w)}. \end{array}$$ (31)

The input neurons encode the actual observable input variables *x*_*j*_s with a population code in order to assess different combinations of input neuron spiking states for every possible input vector **x** = {*x*_1_, *x*_2_, …, *x*_*N*_} from the raw input data to be classified. The state of an input variable *x* is encoded using a group of input neurons, where only one neuron in the group can fire at each time instance to represent the instantaneous value of *x*(*t*). Therefore, together with the total prior probabilities ∑*p*(*k*|**w**) = 1, the Bayesian computation of the network shown in Eq 31 operates under the constraints,

$$\begin{array}{c} \sum_{k=1}^{K}e^{w_{k0}}=1,\;\forall k:\sum_{i\in G_{j}}e^{w_{ki}}=1,\;j=\left(1,2,...,N\right) \end{array}$$ (32)

where *G*_*j*_ represents a set of all possible values that an instantaneous input evidence *x*_*j*_ can have, which is also equivalent to the discretized value of each input variable in the continuous case. This means that an input evidence *x*_*j*_ for a feature *j* of observed data is encoded as a group of neuronal activations *y*_*i*_. If the set of possible value of *x*_*j*_ consists of *m* values *G*_*j*_ = [*v*_1_, *v*_2_, …, *v*_*m*_], the input *x*_*j*_ is encoded using *m* input neurons. Therefore, if input data is given as a *N* (*j* = 1, …, *N*) dimensional vector, the total number of input neurons is *mN*.

#### Synaptic and neuronal plasticities

Synaptic plasticity in the STDP connections (Fig 4) captures both biological plausibility and the computational requirement for Bayesian inference. The LTP part of the STDP curve used follows the shape of EPSPs at the synapses [30], which is similar to biological STDP, in that the backpropagating action potential from a postsynaptic neuron interacts with the presynaptic EPSP arriving at the synapse. The magnitude of the weight update depends on the inverse exponential of the synaptic weight itself to prevent unbounded weight growth. Let us denote the connection weight from the *i*’th input neuron to the *k*’th ensemble layer neuron as *w*_*ki*_, where now *k* indicates the index for the entire layer of ensemble neurons (except the gating network), not the index within each WTA circuit. The synaptic update at time *t* is given by:

$$\begin{array}{c} \Delta w_{ki}\left(t\right)=y_{i}\left(t\right)\cdot c\cdot e^{-w_{ki}}-1 \end{array}$$ (33)

where *y*_*i*_(*t*) is the sum of EPSPs evoked by all presynaptic spikes as in Eq 11, and *c* (which is set to *e*^5^ throughout the experiment) is a constant which determines the upper bound of synaptic weights. The LTD part was set to decrease by a constant amount of 1. Given the EPSP caused by presynaptic spikes, synaptic update occurs only at the moment of a postsynaptic neuron firing, with a certain learning rate. This plasticity rule can exhibit the stimulus frequency dependent behaviour of biological synapses which has been observed in biological STDP experiments [69], where the shape of the plasticity curve (including the traditional hyperbolic curve of the phenomenological model [70, 71]) depends on the repetition frequency of the delayed pairing of pre and postsynaptic stimulations.

In contrast to the logical ITDP model, the SEM ITDP ensemble uses the more biologically realistic ITDP plasticity curve shown in Fig 2 middle (Simplified ITDP curve). The continuous ITDP curve also serves for dealing with the irregular spike trains output by the presynaptic SEM networks, whereas the logical ITDP ensemble model concurrently fires a single spike from each voter. The ITDP plasticity curve is defined as a function of the time difference between two input stimuli using a Gaussian factor. As in the logical ITDP model, the peak LTP at an input time delay of -20ms (where distal input precedes proximal input by 20ms) in biological ITDP is ignored for computational convenience, by assuming that the axon converging on the proximal synapse already has 20ms of conduction delay. Thus the plasticity curve was shifted to have its peak value at zero delay. The change of the ITDP synapse from the *k*th ensemble layer neuron to the *f*th neuron of the final output WTA (see Fig 4) can be written as:

$$\begin{array}{c} \Delta w_{fk}=h_{fk}\left(t\right)\cdot c\cdot e^{-w_{fk}}-1 \end{array}$$ (34)

$$\begin{array}{c} h_{fk}\left(t\right)=\sum_{s_{f}^{G}}\sum_{s_{k}}g\left(t_{f}^{G}-t_{k}\right) \end{array}$$ (35)

$$\begin{array}{c} g(x)=e^{-x^{2}/2\sigma^{2}} \end{array}$$ (36)

where *h*_*fk*_(*t*) is the sum of all synaptic potentiations evoked by the spike time differences between the proximal (from ensemble neurons, *s*_*k*_) and distal (from gating neurons $s_{f}^{G}$) inputs, calculated by the Gaussian function *g*(*x*). The proximal weight *w*_*fk*_ is updated whenever either of the two presynaptic neurons spike. In the same way as the STDP update rule, the ITDP synaptic change is regulated by an inverse exponential dependence on the weight itself and a constant synaptic depression of 1, which results in the simplified ITDP curve shown in Fig 2. The variance of *g*(*x*) was set to *σ*^2^ = 1.5×10^−4^, where the *x* axis represents the spike time difference in seconds.

The self-excitability of the WTA output neurons is modelled in a way that is directly analogous to the plasticity of the synaptic weights. Recalling the membrane potential *u*(*t*) of a SEM circuit neuron in Eq 9, the excitability *w*_*k*0_ of neuron *z*_*k*_ is increased whenever it fires (*z*_*k*_(*t*) = 1) as a function of the inverse exponential of *w*_*k*0_ and is decreased by 1 if not firing (*z*_*k*_(*t*) = 0).

$$\begin{array}{c} \Delta w_{k0}=z_{k}\left(t\right)\cdot e^{-w_{k0}}-1 \end{array}$$ (37)

The update of *w*_*k*0_ is circuit-spike triggered, which means that the excitabilities of all neurons in the WTA circuit are updated if one of the neurons fires. Therefore the value of *w*_*k*0_ converges to satisfy the normalization constraint as a prior probability which is necessary for the above mentioned Bayesian computation.

All the plasticities of STDP, ITDP, and neuronal excitability described above are updated at their corresponding trigger conditions by *w* ← *w* + *η*Δ*w*. The learning rates (*η*) of every individual synapse and excitability are adaptively changed by a variance tracking rule [43] as:

$$\begin{array}{c} \eta_{new}=\frac{\mu (m_{2}-m_{1}^{2})}{1+e^{-m_{1}}} \end{array}$$ (38)

$$\begin{array}{c} m_{1}\leftarrow m_{1}+\eta \left(w-m_{1}\right) \end{array}$$ (39)

$$\begin{array}{c} m_{2}\leftarrow m_{2}+\eta \left(w^{2}-m_{2}\right) \end{array}$$ (40)

where *m*_1_ and *m*_2_ are the first and the second moments of the corresponding learning variable. *μ* is a constant which is globally set to 0.01. The learning rate and moments are updated together whenever the update of a learning variable is triggered.

#### SEM-ITDP experiments

The core (common) numerical details of the SEM-ITDP experiments are as follows: *T*_*present*_ = 40*ms*, *T*_*rest*_ = 40*ms*. During input presentations, one of the two input neurons that encode a pixel state fires with a constant rate of 40Hz.

The common network parameters (used in all experiments) were as follows; *A*_inh_ = 3000, *O*_inh_ = −550, *τ*_inh_ = 0.005sec for neuronal inhibition, *τ*_*s*_ = 0.015sec, *τ*_*f*_ = 0.001sec, *A*_EPSP_ = {*τ*_*s*_/(*τ*_*s*_−*τ*_*f*_)}(*τ*_*s*_/*τ*_*f*_)^*τ*_*f*_/(*τ*_*s*_−*τ*_*f*_)^ for the EPSP kernel. Due to the smaller number of afferent connections to the final WTA than the ensemble layer WTAs, its global inhibition level was shifted (increasing output by giving less inhibition) by an amount proportional to the ensemble size *N*_*E*_ (i.e. related to the number of presynaptic neurons) in order to match the output intensity to those of the ensemble neurons. The inhibition level of the final WTA circuit was set as $A_{\text{inh}}^{\text{f}}=3000-I_{s}$ and $O_{\text{inh}}^{\text{f}}=-550+I_{s}$, with the level of shift *I*_*s*_ = 560 − 4*N*_*E*_.

#### Feature selection using Gaussian distributions

**Fig 16:**
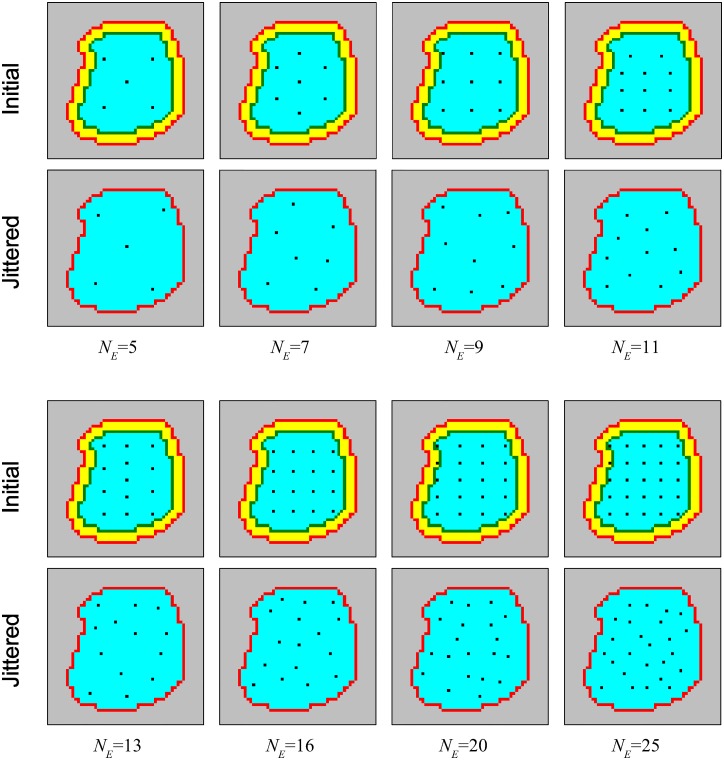
Examples of random Gaussian mean placements for different *N*_*E*_ from the manually designed initial points (black points). The red pixels represent the outer border of the active region of the image, and the yellow pixels represent a forbidden region which is 3 pixels thick. The jittered mean points were restricted to be placed inside the inner region (including the green pixels) which is surrounded by the inner border (green).

The normal Gaussian selection scheme worked by sampling pixels from 2D normal distributions with different means. The distribution function for the *i*th ensemble network was:

$$\begin{array}{c} p_{i}\left(x,y\right)=0.1\times \text{exp}\left(-\frac{{\left(x-\mu_{i}^{x}\right)}^{2}+{\left(y-\mu_{i}^{y}\right)}^{2}}{2\sigma^{2}}\right) \end{array}$$ (41)

with the variance *σ*^2^ = 49. Different means ($\mu_{i}^{x},\mu_{i}^{y}$) for each ensemble WTA were located evenly on the active region of image to cover every region. Although the Gaussian means for each ensemble WTA can be evenly placed simply by using a regular lattice of different sizes on the image, their locations were stochastically generated by a simple optimization procedure in order to reduce any potential bias from a single specific formation of the means on the image (the random elements are also more biologically relevant). In order to reduce the computation time for the optimization, the mean positions were jittered by a small amount around the manually placed initial positions under a certain constraint (Fig 16). The initial mean positions were properly designed to be evenly scattered across the image for each ensemble size in order to prevent any biased placement of the generated mean positions. A simple iterative procedure for the random Gaussian mean placement is as follows:

1. Given the set of initial mean points ($U_{i}^{x},U_{i}^{y}$), *i* = {1, 2, …, *N*_*E*_}, every mean point ($\mu_{i}^{x},\mu_{i}^{y}$) is drawn by randomly jittering the corresponding initial point as: $\mu_{i}^{x}=U_{i}^{x}+\Delta \mu_{x}$ and $\mu_{i}^{y}=U_{i}^{y}+\Delta \mu_{y}$, where Δ*μ*_*x*_ and Δ*μ*_*y*_ are randomly drawn in the range [−*ϵ*, *ϵ*].
2. Repeat 1 until every mean point ($\mu_{i}^{x},\mu_{i}^{y}$) is inside the inner region (which is surrounded by the green pixels as in Fig 16), where the minimum distance *d*_*min*_ between all pairs of mean points satisfies *d*_*min*_ > *δ*.

The parameters *ϵ* and *δ* are set for each ensemble sizes *N*_*E*_ = {5, 7, 9, 11, 13, 16, 20, 25} as: (*ϵ*, *δ*) = {(9, 14), (7, 10), (5, 9), (5, 7.5), (5, 7), (5, 5.5), (5, 4.5), (3, 4.2)}, which were found to allow the optimization process to execute in a reasonable time while producing reasonably evenly distributed mean points.

The stretched Gaussian distribution selected pixel subsets to form a bar shape (at different orientations) as shown in Fig 9 bottom. The probability density function for stretched Gaussian distribution was:

$$\begin{array}{c} p_{i}\left(x\right)=\text{exp}\left(x^{T}\Sigma_{i}^{-1}x\right) \end{array}$$ (42)

where **x** = (*x*, *y*) is a random vector (mean at the origin), and Σ_*i*_ is the covariance matrix (symmetric, positive definite) for the *i*th ensemble member. Each element of the inverse covariance matrix is written as:

$$\begin{array}{c} \Sigma_{i}^{-1}=\left[\begin{array}{cc} a & b\\ b & c \end{array}\right] \end{array}$$ (43)

$$\begin{array}{c} a=\frac{{\text{cos}}^{2}\theta_{i}}{2\sigma_{x}^{2}}+\frac{{\text{sin}}^{2}\theta_{i}}{2\sigma_{y}^{2}}\;b=\frac{\text{sin}2\theta_{i}}{4\sigma_{y}^{2}}-\frac{\text{sin}2\theta_{i}}{4\sigma_{x}^{2}}\;c=\frac{{\text{sin}}^{2}\theta_{i}}{2\sigma_{x}^{2}}+\frac{{\text{cos}}^{2}\theta_{i}}{2\sigma_{y}^{2}}. \end{array}$$ (44)

The variances for the ellipsoids were set to $\sigma_{x}^{2}=4$ and $\sigma_{y}^{2}=625$ identically for all ensemble members (i.e. the pixels practically form a bar shape), except its orientation is rotated by *θ*_*i*_ rad. Starting from *θ*_0_ = 0, the orientations are incremented by *π*/*N*_*E*_ for each successive distribution (i.e. *θ*_*i*_ = *i*.*π*/*N*_*E*_).

## Supporting Information

S1 Text Text and figures giving full details of the methods and results of a validation of the analytic solutions for the initial abstract/simplified ensemble learning model (the voter ensemble model) by numerical simulation. (PDF)
