# Supplementary material for: Unsupervised Learning in an Ensemble of Spiking Neural Networks Mediated by ITDP
Source: PLoS Comput Biol. 2016 Oct 19;12(10):e1005137. doi: 10.1371/journal.pcbi.1005137 (PMC5070787; doi:10.1371/journal.pcbi.1005137)
Supplement: S1 Text — (PDF) [file pcbi.1005137.s001.pdf]

S1 Text: Supporting information for 'Unsupervised Learning in an Ensemble of Spiking Neural Networks mediated by ITDP'

Validation of Analytic Solutions by Numerical Simulation

Yoonsik Shim[1], Andy Phillipides[1], Kevin Staras[2] and Phil Husbands[1]
1. Centre for Computational Neuroscience and Robotics, University of Sussex
2. Neuroscience, School of Life Sciences, University of Sussex

This document gives details of the methods and results of a validation of the analytic solutions for the initial abstract/simplified ensemble learning model (the voter ensemble model) by numerical simulation.

Validation of Analytic Solutions by Numerical Simulation

In order to validate the analytic solutions of the voter ensemble network, we compared its results, as derived in the first part of the Results Section of the main paper, with a numerical simulation that simply iterated through all the underlying equations of the same model. This validation was deemed worthwhile because the simplified analytical model is based on Bernoulli random variables that simulate per sample firing events. The numerical simulation of the model allowed us to check that the long-term trends and statistics matched those predicted by the analytical solutions. The simulation was performed by a two-step process: a learning phase was followed by a measuring phase. Since this initial simplified model is for illustrative purposes (to determine if the ITDP and ensemble learning dynamics are as expected), for convenience and ease of analysis, abstract automatically generated data was used for the simulation. First, we defined the posterior probability tables as in Figure 3 (in the main paper) for every ensemble voter and the gating voter by randomly drawing their values using a certain distribution. After defining the posterior probabilities for an input dataset of size M, the learning phase trains the connection weights using ITDP until they are sufficiently converged by running the system for multiple rounds of dataset presentation. This is followed by the measuring phase which measures the performance of the final voter using these fixed weights. The performance of a single voter was measured using normalised conditional entropy (NCE) [1], which is suitable for measuring the performance of a multi-class discrimination task where the explicit relation between the neuronal index and the corresponding class is unavailable. NCE has a value in the range $0 \leq \text{NCE} \leq 0.5$, with lower conditional entropy indicating that each neuron fires more predominantly for one class, hence giving better performance (See Methods for the details of the simulation procedure and the NCE calculation).

Comparison with Numerical Simulation

Both the learning and measuring phases in the numerical simulation were run for $T = M \times R$ steps, where R is the number of rounds of dataset presentation which was set to 10 or 100, depending on the experiment. Using 4 input classes ($N_C = 4$) the total number of input samples was set to $M = 400$ which consists of 100 samples for each class. The pre-determined firing probabilities of the ensemble and the gating voter can be set arbitrarily under the constraint $\sum_{i=1}^{N_C} p(m_i^j|x) = 1$. In order to easily test and understand the broad picture of the ensemble behaviour (as well as to aid performance visualisation), let us represent the system by a few

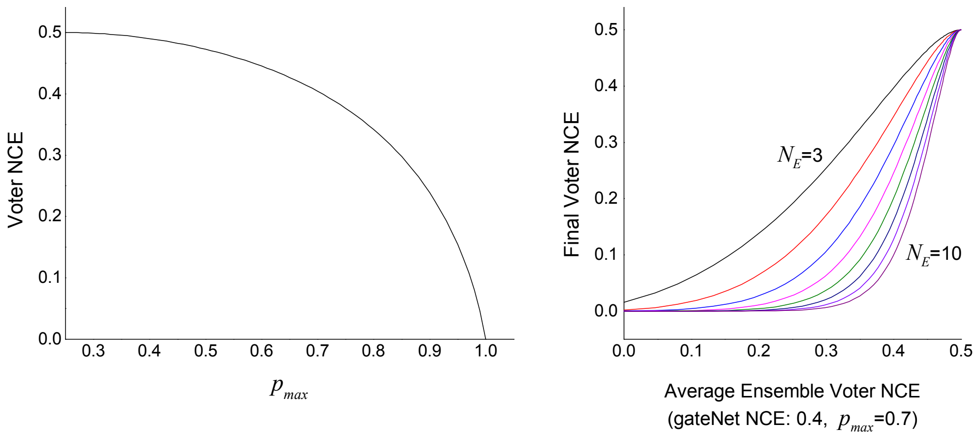

Figure S1.1. Illustrative descriptions of the changes of the analytically calculated NCEs for a single voter and the final voter under various values of P_{max} and ensemble sizes N_E. For illustration purpose, the neuron firing probabilities of a voter were fixed to P_{max} for the dominant firing neuron and r for the rest (See text for details). Average ensemble voter NCE was simply controlled by using identical parameters for all ensemble voters. (Left) The NCE of a voter vs. P_{max}. (Right) Bivariate plots of the final voter NCEs vs. average ensemble NCEs with different ensemble sizes $3 \leq N_E \leq 10$. The gating voter NCE is fixed to 0.4 by setting $P^G_{max} = 0.7$.

parameters describing the presynaptic voters. Since the final voter performance is affected directly by the performance of the ensemble and gating voters, and therefore cannot be objectively measured independently of them, we consider the three key factors to be: the overall performance of the ensemble voters, the gating voter performance, and the ensemble size. Controlling the overall performance of the ensemble voters is achieved by using statistically identical voters for the entire ensemble. The performance of a voter can be adjusted by varying a parameter which changes all of its firing probabilities as follows.

For $N_C = 4$, the mean firing probabilities of the neurons in each voter for the samples from each class can be considered as a 4×4 matrix where each column is set to the average firing probabilities of a voter for the samples in the corresponding input class. We can see that the voter performance is maximum (lowest NCE) when this matrix is the identity matrix. Also the performance of a voter is analytically the same under the permutation of rows (i.e. switching neuron indices) as can be seen in Equations 27 and 28 (main paper). Thus in order to easily assign various performance values to the voters, we defined a parameter $\frac{1}{N_C} \leq P_{max} \leq 1$ such that every diagonal element of the matrix is set to P_{max}, and all other elements are set equally to $r = (1 - P_{max})/(N_C - 1)$. The probability matrix of a voter for $N_C = 4$ is written as:

$$\begin{pmatrix} P_{max} & r & r & r \\ r & P_{max} & r & r \\ r & r & P_{max} & r \\ r & r & r & P_{max} \end{pmatrix}, \quad r = \frac{1 - P_{max}}{3}, \quad \frac{1}{4} \leq P_{max} \leq 1 \tag{S1.1}$$

This probability matrix can be used to design a voter such that its NCE has the desired value. The simple case would be to assign these class-dependent firing probabilities identically for all samples within the corresponding class, which results in the NCE ranging from 0.5 (worst) to 0 (best) by setting P_{max} accordingly. Using this setting, an initial illustrative picture of voter ensemble behavior is shown in Figure S1.1 which describes the analytically calculated voter NCE for different P_{max} values and the final NCEs of combined output for different average ensemble NCEs and its sizes. However, having identical firing probabilities for entire sample subsets is not realistic. Thus we assign the firing probabilities of every sample for each subsets randomly by using the elements of the above probability matrix as the means of normal distributions

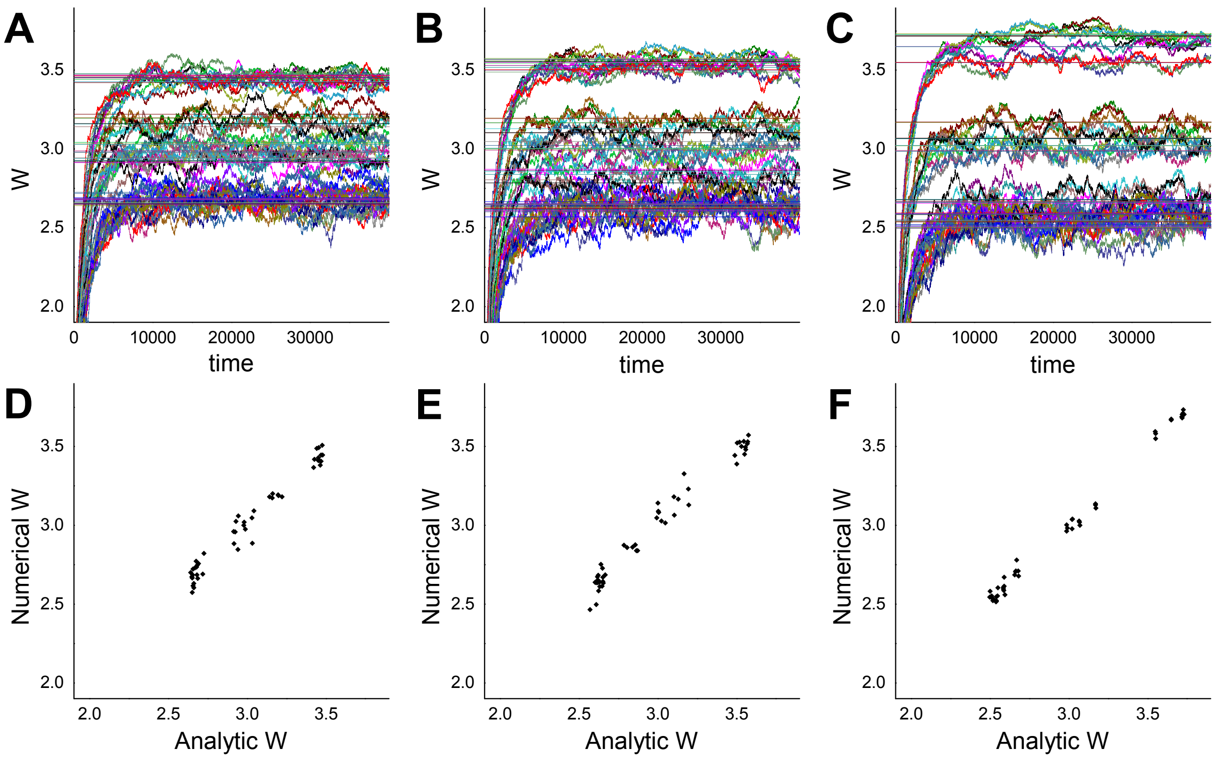

Figure S1.2. Time evolution of weights by numerical ITDP and their analytic solution for different ensemble performances (48 connections run for 40000 time steps (100 rounds with 400 samples), $N_C = 4$ and $N_E = 3$). (A-C) The horizontal lines show the expected level of convergence calculated from the analytic solution. (D-F) The final value of numerical weights vs. analytic weights, each corresponds to A-C. The analytic solution matches the weight values converged by numerical simulation. In order to see the distribution of weight values under higher sample dependent variability of ensemble statistics, the sample-wise firing probabilities of each voter were drawn uniformly randomly in the range $P_L < P_{max} < P_H$ (for the dominantly firing neurons for corresponding classes) and $0.2R < r_{1,2} < 0.8R$ (the rest of probabilities) where $R = (1 - P_{max})/(N_C - 2)$. The gating voter firing probabilities were similarly set in the range $0.5 < P_{max} < 0.6$. The min/max values (P_L, P_H) for P_{max} were set as: (A,D): (0.3,0.9), (B,E): (0.5,0.9), (C,F): (0.7,0.9).

with certain variances. For example, when $N_C = 4$, the firing probabilities for four neurons for a sample from class 1 are set as (p, r_1, r_2, r_3) where $p = \mathcal{N}(P_{max}, \sigma_p^2)$, $r_{1,2} = \mathcal{N}(r, \sigma_r^2)$, and $r_3 = 1-(p+r_1+r_2)$. Probability assignment using uniform distributions is also possible to assess wider diversity on the posterior probabilities of samples by defining the range as $U(P_L, P_H)$.

Using these parameter settings, the ensemble system was tested with different performances of the ensemble voters and the gating voter. First the weight convergence by ITDP was examined by comparing the analytic solution of the expected values of weights and the weights learnt by numerical simulation. Figure S1.2 shows clear separation of weight values into groups according to the discriminability of the ensemble voters, indicating that the ITDP learning reflects the history of presynaptic voter behaviours, which influences the final voter performance. After learning with sufficient guidance from the gating voter, the decision from the better performing ensemble neurons influences the final voter output more by developing relatively stronger weights than the other neurons. Thus the spike from one strongly weighted synaptic projection can overwhelm several other weakly weighted 'wrong' decisions, achieving the weighted vote learnt from the previous history of ensemble behaviour (exactly the behaviour we desire in this kind of ensemble learning). Plotting numerical vs. analytic weights, as in Figure S1.2 D-F, shows that

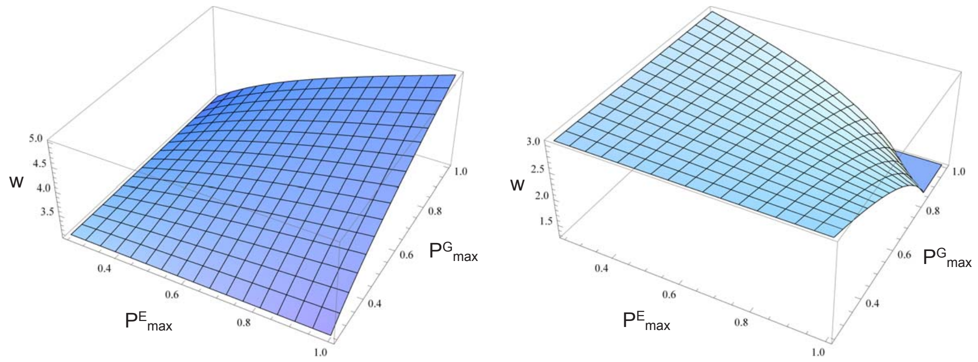

Figure S1.3. Analytic illustration of outgoing connection weights from an ensemble neuron to final voter neurons, depicted on (P^E_{max}, P^G_{max}) space. (Left) Weight delivering the right class. (Right) The other weight which does not match the current presented class. See text for further details.

the two solutions match well, validating our analytic formulation of expected weight values, and demonstrates that the system performs very well under appropriate parameter settings.

The main role of the gating voter is to guide the spike-evoked EPSPs from the ensemble neurons which represent the same class onto one of the postsynaptic neurons of the final voter via topographic distal signals. The analytic model allows us to, for example, easily gain insight into the distribution of the outgoing weights from an ensemble neuron to each of final voter neurons. For tractability, let us assume that each voter neuron has a constant firing probability for all samples in the same class. Using the firing probabilities of presynaptic voters defined by the diagonal matrices as in Equation S1.1, the probabilities of the ensemble voter and the gating voter can be defined by the parameters P^E_{max} and P^G_{max} respectively. Assuming the probabilities of input class presentations ($p(c_n)$) are all the same, the analytic solution of the weight from one of the ensemble voter neurons (m_i) to the final voter neuron f_k can be rewritten using class-conditional probabilities $p(m|c)$ as

$$w_{ki} = \log(a) - \log\left(\frac{\sum_{n=1}^{N_C}\{p(m_i|c_n) + p(g_k|c_n)\}}{\sum_{n=1}^{N_C} p(m_i|c_n)p(g_k|c_n)} - 1\right) \quad \text{(S1.2)}$$

Setting $a = e^5$, $N_C = 4$, $N_E = 3$, and substituting $p(m_i|c_n)$ and $p(g_k|c_n)$ for the elements of the corresponding 4×4 diagonal probability matrices as in Equation S1.1, with parameters P^E_{max} and P^G_{max} gives:

$$w_{ki} = \begin{cases} 5 - \log\left(\frac{5+B}{1-B}\right), & \text{if} \quad k = 1 \\ 5 - \log\left(\frac{16-B}{2+B}\right), & \text{otherwise} \end{cases} \quad \text{(S1.3)}$$

$$B = P^E_{max} + P^G_{max} - 4P^E_{max}P^G_{max}. \quad \text{(S1.4)}$$

We can see that the most enhanced connection weight from the presynaptic neuron m_i to the postsynaptic neuron f_k ($i = k$ in the current setting) has an inverse relationship with the rest of the outgoing weights (connections to f_k where $i{\neq}k$) in (P^E_{max}, P^G_{max}) space. Figure S1.3 shows (when $i = 1$) that a low P^G_{max} (i.e. low gating voter performance) results in less difference between outgoing weights from the presynaptic neuron m_1, hence giving equal EPSPs to all postsynaptic neurons (i.e. $w_{11} = w_{21} = w_{31} = w_{41}$, resulting in no capability of weighted voting). The same holds for the ensemble voter parameter (P^E_{max}). Therefore, in our ensemble voter system, both the gating voter and at least one ensemble voter must have positive discriminability (NCE<0.5) in order to learn to perform weighted voting.

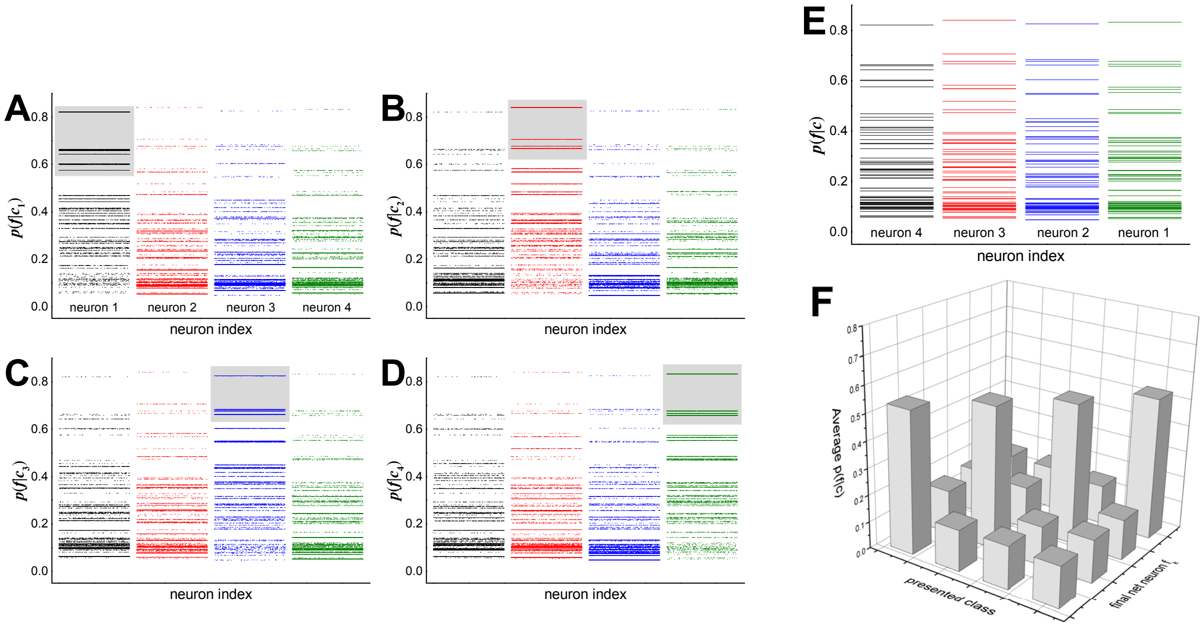

Figure S1.4. Numerical simulation of final voter firing probabilities for each class presentation after learning ($N_C = 4$, $N_E = 3$). The sample-wise firing probabilities of voter neurons were set by a normal distribution of variance 0.1, with the means $P^E_{max} = 0.5, 0.6, 0.7$ for each ensemble voter, and $P^G_{max} = 0.6$ for the gating voter. (A-D) Each plot shows the time course of the firing probabilities of each final voter neuron for different class presentations. Each coloured plot shows the time span $0 \leq t \leq 10000$, where the highest firing probabilities of each final voter neuron appear densely plotted at the corresponding class presentations through learning, as indicated by the grey boxes. (E) Analytic solution of the possible values of each final voter neuron firing probabilities, obtained from the same numerically learnt weights as in A-D. (F) The average firing probabilities of each neuron during each class presentation (the average of each coloured value in the left graphs).

The performance of the final voter depends on the performances of every voter in the ensemble, the gating voter, and the ensemble size. The combined output of the ensemble voter network can be understood by measuring the long-term behavior of the momentary output of the final voter. Figure S1.4 illustrates an example of the final voter probabilities iteratively calculated using the numerical simulation (with fixed weights after learning) with $N_C = 4$, $N_E = 3$. The firing probabilities of voters for each sample was randomly set by a normal distribution with variance 0.1, where the means were set by the parameters $P^E_{max} = (0.5, 0.6, 0.7)$ for each ensemble voter and $P^G_{max} = 0.6$ for the gating voter. The EPSP for the final voter neuron was calculated simply by using the weighted sum of presynaptic spikes, resulting in $4^3 = 64$ possible states of the presynaptic spikes. Thus the maximum number of probability values that each final voter neuron can have is 64, where their individual values depend on the synaptic weights (Figure S1.4 E). At each time instance, the firing probability of each final voter neuron belongs to one of the corresponding set of values, only differing by their frequency of appearance (as shown in Figure S1.4 A-D). The average firing behaviour (Figure S1.4 F) shows that each neuron dominantly fires for a corresponding class, which indicates that the ensemble network successfully achieved weighted voting.

Figures S1.5 shows the relationship between the final voter performance and the presynaptic voter performances, indicating the bidirectional dependence both on the ensemble and the gating voters, as well as illustrating the close correspondence of the analytic solutions and numerical simulations of the logical model. The sample-wise neuron firing probabilities of all voters in the

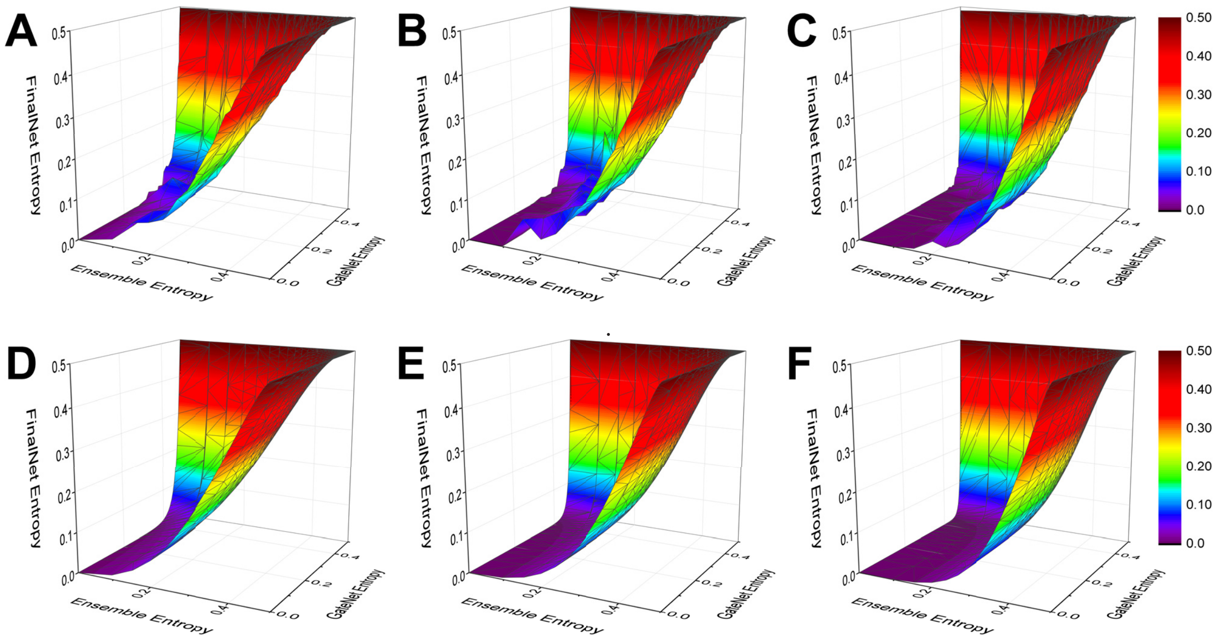

Figure S1.5. Analytic (A-C) and numerical (D-F) long-term performances of the final voter vs. ensemble and gating voters ($N_C = 4$). The parameters for the ensemble (P^E_{max}) and the gating (P^G_{max}) voters vary in the range $[0.25, 1.0]$ with the interval of 0.025. Each column shows the result using different ensemble sizes, where (A,D) $N_E = 3$, (B,E) $N_E = 4$, and (C,F) $N_E = 5$.

ensemble are set by sampling from identical gaussian distributions of $\mathcal{N}(P^E_{max}, 0.05)$ in order to easily assign various overall average ensemble performances (and P^G_{max} for the gating voter). The results show a very good match between the numerical simulations and their analytic solutions over the different combinations of the ensemble and gating voter parameters ($0.25 < P^E_{max}, P^G_{max} < 1.0$). The pairs of graphs in each column show the effect of increased ensemble size, which improves the weighted vote performance as analytically predicted in Figure S1.1-right.

These validation tests showed that the logical model of a spiking voter ensemble system and its analytic solutions are capable of performing efficient spike-based weighted voting, driven by ITDP, and gives us important insights into how that is achieved. They also demonstrated how the seemingly complex network of interactions between stochastic processes within a population of voters can be effectively described by a series of probability metrics.

References

1. Nessler B, Pfeiffer M, Buesing L, Maass W. Bayesian Computation Emerges in Generic Cortical Microcircuits through Spike-Timing-Dependent Plasticity. PLOS Computational Biology. 2013;9(4):e1003037.
